# Epidemiological Characteristics and Genetic Diversity of Chicken Infectious Anemia Virus (CIAV) in Guangdong Province, China

**DOI:** 10.3390/vetsci12100972

**Published:** 2025-10-10

**Authors:** Yongkun Lu, Wenjun Li, Yingying Liu, Junjie Lin, Haojian Luo, Yiqiao Wang, Fenfen Xu, Zhaoping Liang, Kun Mei, Shujian Huang

**Affiliations:** 1 School of Animal Science and Technology, Foshan University, Guangyun road, Shishan Town, Nanhai District, Foshan 528225, China; 15013320178@163.com (Y.L.); lwj517612@163.com (W.L.); yyliu0603@163.com (Y.L.); 15918059606@163.com (J.L.); 18664820794@163.com (H.L.); wyq1498@foxmail.com (Y.W.); 2 Guangdong Hua Sheng Biotechnology Co., Ltd., 1902, Building 14, Xincheng Entrepreneurship Center, Zengcheng Low-carbon Headquarters Park, No. 400, Xincheng Avenue, Zengcheng District, Guangzhou 511300, China; xufenfen@huassw.com; 3 College of Veterinary Medicine, South China Agricultural University, Guangzhou 510642, China; 13922159668@163.com

**Keywords:** CIAV, serological survey, phylogenetic analysis, MSB1 cells, virus isolation

## Abstract

**Simple Summary:**

Chicken infectious anemia virus (CIAV) is an immunosuppressive pathogen that causes considerable economic loss to the global poultry industry. Despite its importance, limited epidemiological data are available for Guangdong Province, China. In this study, we developed a PCR-based assay to improve the accuracy of CIAV detection. A total of 105 tissue samples and 786 serum samples were collected from poultry farms across nine major cities in Guangdong between July 2018 and March 2022. Isolated CIAV strains were further characterized for cellular pathogenicity and genetic evolution. The findings are intended to provide insights that support molecular epidemiological surveillance of CIAV in Guangdong Province.

**Abstract:**

Chicken infectious anemia virus (CIAV) causes immunosuppression in poultry, leading to substantial global economic losses through both vertical and horizontal transmission. Since 2014, frequent outbreaks have been reported in southern China; however, the epidemiology of CIAV in Guangdong Province remains poorly defined. Between July 2018 and March 2022, we collected 105 tissue samples and 786 serum samples from poultry in nine cities. PCR/qPCR assays targeting the VP1 gene confirmed CIAV infection, and positive tissues inoculated into MSB1 cells yielded four isolates (GDHZ1, GDHZ2, GDJM, GDLF). Phylogenetic analysis demonstrated that GDHZ1, GDJM, and GDLF clustered within clade A1, whereas GDHZ2 belonged to clade A2. All isolates shared glutamine (Q) at position 394, together with virulence-associated amino acid signatures (75V, 89T, 125L, 139K, 141Q, 144E). Serological testing indicated a high prevalence, with 627 of 786 samples positive (79.77%). The relatively low proportion of virus-positive tissues and successful isolations may reflect viral tropism or limitations in detection sensitivity. These findings enhance understanding of CIAV molecular epidemiology in Guangdong and provide evidence to inform surveillance, vaccination strategies, and control measures.

## 1. Introduction

Chicken infectious anemia virus (CIAV) is a globally distributed immunosuppressive pathogen of poultry, first identified in Japan and subsequently reported in China in 1992, from where it spread nationwide [1,2].

Epidemiological surveys have consistently reported high prevalence rates of CIAV in southern China. During 2004–2005, up to 87% of chickens sampled from live bird markets tested positive [3]. Recent investigations conducted between 2015 and 2018 reported positivity rates of 17.1% in southern China [4]. In Guangdong Province, approximately 33% (91/277) of farmed chickens tested positive during 2016–2017 [5]. Infected chickens typically exhibit anemia, growth retardation, and increased mortality, but the most critical feature is immunosuppression, which predisposes flocks to secondary infections and reduces the efficacy of vaccination against other pathogens [6,7]. These findings underscore CIAV as a major threat to poultry health and highlight considerable regional variation in prevalence.

CIAV belongs to the genus Gyrovirus within the family Parvoviridae. It is a small, non-enveloped virus with a single-stranded circular DNA genome encoding three open reading frames. Among them, the capsid protein VP1 is the principal immunogenic protein and a key determinant of viral pathogenicity and evolution [8,9,10]. Because of its relatively conserved sequence combined with functionally important variation, VP1 has become the primary target for molecular detection and phylogenetic studies.

In recent years, frequent CIAV outbreaks have been reported in southern China, yet comprehensive epidemiological data from Guangdong Province remain scarce. Previous studies indicate that CIAV strains are divided into two major clades (A and B), with Clade A currently predominant and showing an increasing prevalence trend in China [11,12]. Against this backdrop, the present study integrated serological surveillance, PCR and quantitative PCR (qPCR) assays targeting the VP1 gene (Patent number: CN113897356A), virus isolation, and phylogenetic analysis to investigate both the prevalence and genetic diversity of CIAV in Guangdong. The findings aim to fill a critical knowledge gap and provide valuable insights for molecular epidemiological surveillance and disease control strategies in the poultry industry.

## 2. Materials and Methods

### 2.1. Cells

Chicken lymphoblastoid cell line MSB1 (from laboratory stock) was used for propagation and infectivity titration of CIAV. Cells were cultured in RPMI 1640 medium (Gibco, Waltham, MA, USA) supplemented with 10% fetal bovine serum (Gibco, Waltham, MA, USA) and 1% penicillin-streptomycin (Hyclone, Logan, UT, USA), at 37 °C under 5% CO_2_ atmosphere.

### 2.2. Sample Collection and Processing

Between June 2018 and September 2019, a total of 105 thymus samples were collected from broiler farms in Lufeng, Foshan, Huizhou, Qingyuan, Jiangmen, Zhuhai, and Shantou. Birds were selected based on suspected CIAV infection, with clinical signs such as lethargy and growth retardation. In contrast, 786 serum samples were randomly obtained from apparently healthy, non-vaccinated chickens across 23 farms in Guangzhou, Foshan, Zhanjiang, Huizhou, Qingyuan, Jiangmen, Zhuhai, Zhongshan, and Shantou between July 2018 and March 2022.

Thymus tissues were rinsed twice with cold sterile phosphate-buffered saline (PBS), homogenized, and centrifuged at 12,000 rpm for 10 min at 4 °C. Serum samples were treated with 1% streptopenicillin (Sigma-Aldrich, St. Louis, MO, USA) and stored at −80 °C until analysis.

#### 2.2.1. Sampling Strategy

The sampling scheme was designed to balance epidemiological coverage with practical considerations. In regions without overt clinical signs, only serum samples were collected, whereas in outbreak-suspected farms both serum and thymus samples were obtained to enable serological and molecular analyses. Thymus samples were exclusively collected in Lufeng. Sample sizes were calculated using standard prevalence survey formulas to ensure adequate statistical power for estimating CIAV prevalence and supporting viral characterization.

#### 2.2.2. Data Analysis

For each city, the number of CIAV-positive samples was summarized, and prevalence estimates with 95% confidence intervals (CI) were calculated using RStudio (v4.5.1). Statistical significance was defined at *p* < 0.05.

### 2.3. Detection of CIAV Antibodies in Serum Samples

A total of 786 chickens without prior CIAV vaccination were randomly selected for blood collection from 23 breeding farms. Blood was drawn into sterile vacutainer tubes containing EDTA (Beijing Labgic Technology Co., Ltd., Beijing, China) as an anticoagulant and gently inverted several times to ensure adequate mixing. Samples were centrifuged at 3000 rpm for 10 min at room temperature, and the resulting sera were carefully collected and stored at −20 °C until analysis.

All serum samples were tested for CIAV antibodies using a commercial antibody-blocking ELISA kit (IDEXX Laboratories, Inc., Westbrook, ME, USA), following the manufacturer’s instructions.

### 2.4. Sample DNA Extraction and Virus Identification

DNA was extracted from thymus samples using AxyPrep Body Fluid Viral DNA/RNA Miniprep Kit (Axygen Biosciences, Union City, CA, USA) following the manufacturer’s instructions. DNA quality and quantity were determined using a spectrophotometer, and samples were stored at −20 °C until further use.

qPCR was performed to detect CIAV by targeting the VP1 gene using SYBR^®^ Green Premix Pro Taq HS qPCR Kit (Takara Bio Inc., Shiga, Japan). The qPCR amplification procedure was performed under the following thermal-cycling conditions: 3 min at 95 °C, followed by 40 cycles of 95 °C for 5 s and 55 °C for 15 s, 68 °C for 20 s, then 95 °C for 30 s, 65 °C for 30 s and 95 °C for 30 s. The primer sequences used in this study are listed in Table 1.

CIAV detection was achieved using a PCR assay targeting the VP1 gene. The assay employed two sets of specifically designed primers, which could amplify the complete coding sequences of all three ORFs within VP1 (VP1, VP2, VP3). PCR analysis was performed using Premix Taq™ (Takara Bio Inc., Shiga, Japan). Thermocycling conditions were as follows: 4 min denaturation step at 94 °C, followed by 30 cycles of 30 s at 94 °C, 30 s at 53 °C, and 45 s at 72 °C with a 10 min elongation step at 72 °C. Primer sequences used for PCR analysis are presented in Table 2.

### 2.5. Isolation of the Virus

For CIAV detection, specific primers targeting the VP1 gene were designed and employed in a PCR assay. Subsequently, virus isolation was attempted from PCR-positive thymus samples. The thymus tissue suspension made in the above steps was inoculated into cells and embryonated chicken eggs.

MSB1 cells were maintained in suspension culture supplemented with 10% fetal bovine serum (FBS) and 1% penicillin–streptomycin (Sigma-Aldrich, St. Louis, MO, USA). Freshly prepared cultures containing 2.5 × 10^5^ cells/mL were seeded into six-well plates. Cells were inoculated with 400 μL per well of thymus homogenates at a 1:5 (*v*/*v*) dilution. Infected cultures were monitored regularly for the development of cytopathic effects (CPEs) [13].

### 2.6. Indirect Immunofluorescence Assays (IFA)

Since minimal or no toxicity was observed in MSB1 cells infected with CIAV, indirect immunofluorescence assays (IFA) were employed to determine the infection rate.

The detailed procedure was as follows: infected cells were fixed with 4% paraformaldehyde (Solarbio, Beijing, China) for 10 min, followed by a 30 min blocking step with 1% Bovine Serum Albumin (BSA). Cells were then incubated with positive sera (prepared in our laboratory) diluted 1:500 for 1 h and washed thrice with PBS. Cells were further incubated with goat anti-chicken IgY (Abcam, Cambridge, UK) diluted 1:1000 for 1 h in the dark. Fluorescing MSB1 cells were observed, counted, and photographed under an inverted fluorescence microscope [14].

### 2.7. Sequence Analysis

The full-length nucleotide sequences of the CIAV genome were obtained by Sanger sequencing (first-generation sequencing). Sequence data were assembled and analyzed using DNAman software (v9.0) (Lynnon Biosoft, San Ramon, CA, USA), and full-length genomes were reconstructed. Sequences were manually edited with BioEdit (v7.12) (Tom Hall, Ibis Therapeutics, Carlsbad, CA, USA) and subsequently used as queries in BLAST(v2.16.0) NCBI, Bethesda, MD, USA) searches against the GenBank database to identify homologous CIAV sequences.

Multiple sequence alignments were performed with the ClustalX algorithm, and phylogenetic trees were constructed using the neighbor-joining method implemented in MEGA X (v10.1.8). The Tamura–Nei model was applied, and node reliability was assessed with 1000 bootstrap replicates. Gene-level analyses of CIAV isolates were conducted using Computational Life Sciences (CLC) Sequence Viewer (v8.0) and the MegAlign module of the DNASTAR package (v11.0) (DNASTAR, Inc., Madison, WI, USA). Protein structure predictions for the VP1 protein were generated with ESMFold (https://wemol.wecomput.com/ui/#/frontend/workflow/workflow-modules), ULR (accessed on 6 June 2024) [15].

## 3. Results

### 3.1. CIAV Serological Detection

Between 2018 and 2022, a total of 786 serum samples were collected from breeding and broiler chicken farms across nine regions in Guangdong Province, including Guangzhou, Foshan, Zhanjiang, Huizhou, Qingyuan, Jiangmen, Zhuhai, Zhongshan, and Shantou. Of these samples, 627 tested positive for Chicken Infectious Anemia Virus (CIAV) antibodies, yielding an overall positivity rate of 79.77% (627/786). CIAV antibodies were detected across all nine sampled regions. Notably, Zhanjiang and Shantou exhibited the highest CIAV antibody positivity rates, each reaching 100%. In contrast, Zhongshan demonstrated the lowest positivity rate at 38.46% (20/52). The positivity rates in the remaining regions were as follows: Guangzhou at 92.16% (47/51), Foshan at 84.56% (115/136), Huizhou at 68.37% (67/98), Qingyuan at 85.37% (70/82), Jiangmen at 72.22% (117/162), and Zhuhai at 89.15% (115/129). On average, the CIAV antibody positivity rate across these regions exceeded 60% (Table 3).

### 3.2. PCR-Based CIAV Detection

A qPCR and PCR assay targeting the CIAV VP1 gene was developed and detected for 105 thymus samples collected from broiler farms in 7 different regions of Guangdong Province. The suspected CIAV-positive thymus samples were then sequenced for verification.

**Figure 1:**
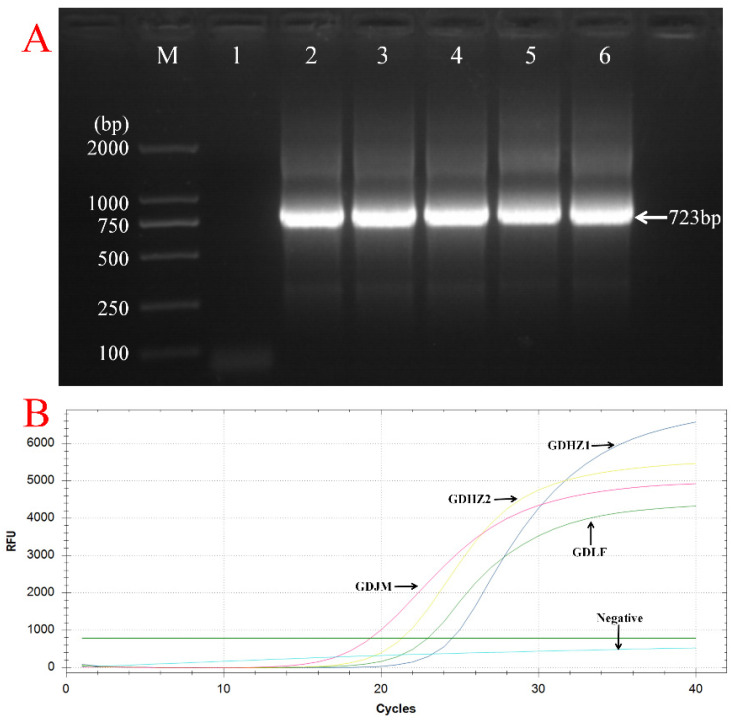
Detection of CIAV in thymus samples using PCR-based assays. (**A**) PCR amplification of the CIAV VP1 gene from selected thymus tissues. Lane M: 2000 bp DNA marker; Lane 1: negative control; Lane 2: positive control; Lane 3: CIAV-GDJM (723 bp); Lane 4: CIAV-GDLF (723 bp); Lane 5: CIAV-GDHZ1 (723 bp); Lane 6: CIAV-GDHZ2 (723 bp). (**B**) Quantitative PCR (qPCR) detection of the CIAV VP1 gene in thymus tissue samples.

The results of qPCR and PCR showed that 4 out of 105 thymus samples were positive for CIAV, and the results of the two methods were consistent (Figure 1, Table 4). Additionally, sequencing of the amplified products confirmed that CIAV infection was present in these thymus samples.

### 3.3. CIAV Isolation in MSB1 Cells

**Figure 2:**
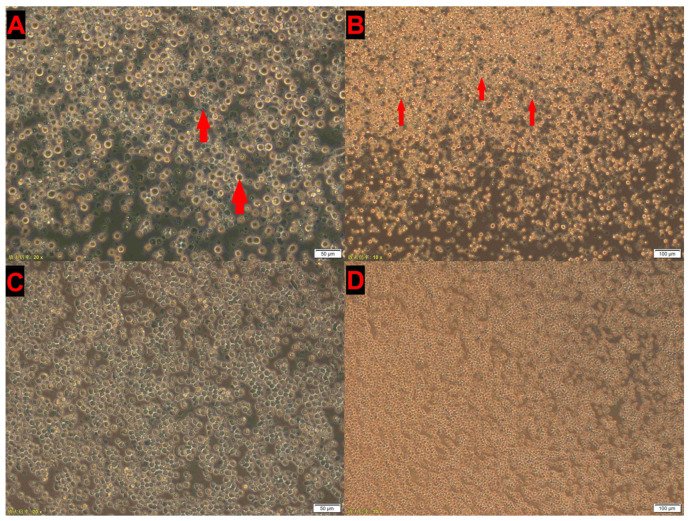
Cytopathic effects (CPE) of CIAV-GDJM in MSB1 cells after the sixth passage. Characteristic CPE, including cell swelling, fragmentation, and accumulation of debris, are indicated by red arrows (red arrows). (**A**) Infected cells at 200× magnification; (**B**) infected cells at 100× magnification; (**C**) uninfected control cells at 200× magnification; (**D**) uninfected control cells at 100× magnification. Notes: The text in the bottom left corner of the figure represents “10× objective magnification of the microscope” and “20× objective magnification of the microscope” respectively.

Primers targeting the VP1 gene (this study) were used for CIAV detection. PCR- and qPCR-positive thymus suspensions were pre-treated and subsequently inoculated into MSB1 cells. During continuous passages, characteristic cytopathic effects (CPEs), including cell swelling, fragmentation, and accumulation of cellular debris, were observed. After five to six serial passages, four CIAV isolates were successfully obtained, all displaying typical CPE under microscopic examination (Figure 2).

### 3.4. IFA Detection

**Figure 3:**
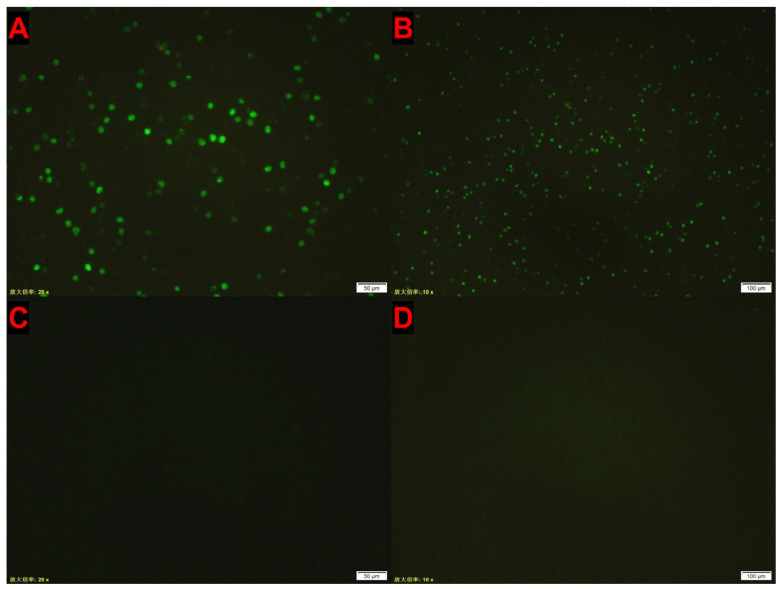
Indirect immunofluorescence assay (IFA) of MSB1 cells infected with CIAV-GDJM. Green fluorescence was detected in the infected group but absent in the control group. (**A**) Infected cells at 72 h post-infection, 200× magnification; (**B**) infected cells at 72 h post-infection, 100× magnification; (**C**) uninfected control cells at 72 h, 200× magnification; (**D**) uninfected control cells at 72 h, 100× magnification. Notes: The text in the bottom left corner of the figure represents “10× objective magnification of the microscope” and “20× objective magnification of the microscope” respectively.

MSB1 cells were subjected to IFA following infection with CIAV-GDJM for 72 h and after six blind passages. Distinct green fluorescence was observed in the infected group (Figure 3A,B), whereas no fluorescence was detected in the uninfected control group (Figure 3C,D).

### 3.5. Genetic Evolution Analysis of CIAV Isolates

**Figure 4:**
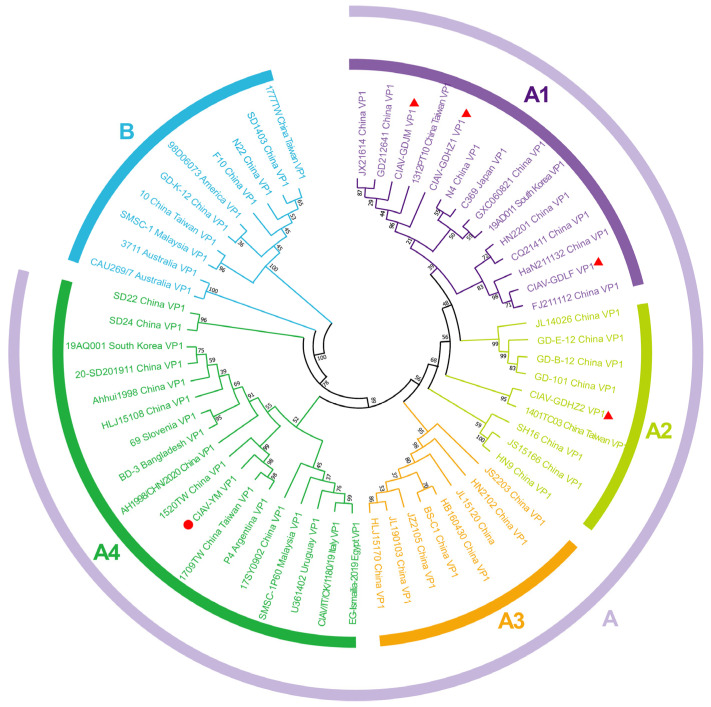
Phylogenetic analysis of the VP1 gene nucleotide sequences of four CIAV isolates. The tree is divided into two major clades (A and B), with Clade A further subdivided into four subgroups (A1–A4). The four isolates obtained in this study are indicated by circles, while vaccine strains are indicated by triangles. Bootstrap values (1000 replicates) are shown at key nodes, and the scale bar represents the number of substitutions per site.

A phylogenetic tree was constructed using the Neighbor-Joining (NJ) method with VP1 nucleotide sequences from CIAV isolates and reference strains. The analysis identified two main groups, Group A and Group B. Group A was further divided into four distinct subgroups: A1, A2, A3, and A4, (Figure 4). Subgroups A1 and A2 predominantly comprised southern China, Japan, and Korea strains. Specifically, CIAV isolates GDJM, GDHZ1, and GDLF were categorized within subgroup A1, with CIAV-GDJM and CIAV-GDHZ1 forming a branch with the Taiwan strain 1312PT10 (KY888915). The Taiwan strain 1401TC03 (KY888918) and the CIAV-GDHZ2 isolate were classified within subgroup A2. CIAV strains predominantly from northern China were grouped under subgroup A3, while strains from eastern China, southern Eurasia, and southern Africa were categorized within subgroup A4. The clustering of the commercial vaccine strain CIAV-YM within subgroup A4 alongside the isolated field strains reinforces the notion that all four viruses under investigation are likely of wild-type origin.

### 3.6. Amino Acid Sequence and Structure Analysis of CIAV Isolates

Our analysis of the VP1, VP2, and VP3 genes in four CIAV isolates and 55 reference sequences obtained from GenBank revealed nucleotide similarities ranging from 98% to 99.3% for the VP1 gene. The VP1 protein nucleotide similarities varied between 94.1% and 99.8%, with strains N22 (Shandong, China), SD1403 (Shandong, China), F10 (Shandong, China), 1777TW (Taiwan, China), and 10 (Taiwan, China) exhibiting lower similarities of 94.1% to 94.9%. Notably, isolate GDJM and the Chinese Shandong strain N22 (KU845734) displayed a nucleotide similarity of 94.1%. At the global scale, the VP1 amino acid similarity varied between 94.3% and 99.0%, with strains SMSC-1 (Malaysia), CAU269/7 (Australia), 3711 (Australia), and 98D06073 (USA) displaying the lowest similarities, ranging from 94.6% to 95.3%. Particularly noteworthy was the finding that isolate GDJM and Australia’s CAU269/7 (AF227982) exhibited the lowest similarity at 94.6%.

The four CIAV isolates demonstrated nucleotide similarities ranging from 99.5% to 100% for the VP2 protein, with domestic reference strains exhibiting similarities of 98.5% to 100%, and international reference strains showing similarities of 98.8% to 100%. Additionally, CIAV isolates displayed nucleotide identities of 99.7% to 100% for the VP3 protein, domestic reference strains showed 98.6% to 100%, and international reference strains displayed identities of 98.4% to 100%.

The VP1, VP2, and VP3 genes of four CIAV isolates were examined utilizing CLC Sequence Viewer and MegAlign software (v11.0) from the DNA Star package. The analysis revealed six unique amino acid substitutions in each isolate, with no detectable nucleotide insertions or deletions. The isolates exhibited 11 amino acid substitutions, with eight occurring in VP1, one in VP2, and two in VP3. The VP1 gene of CIAV harbors crucial amino acid residues linked to virulence, as evidenced by the presence of specific amino acid combinations (75V, 89T, 125L, 139K, 141Q, 144E, and 394Q) in the four examined isolates. Furthermore, two unique amino acid substitutions, T265A and D281G, were discovered in the CIAV-GDJM strain (Table 5).

**Figure 5:**
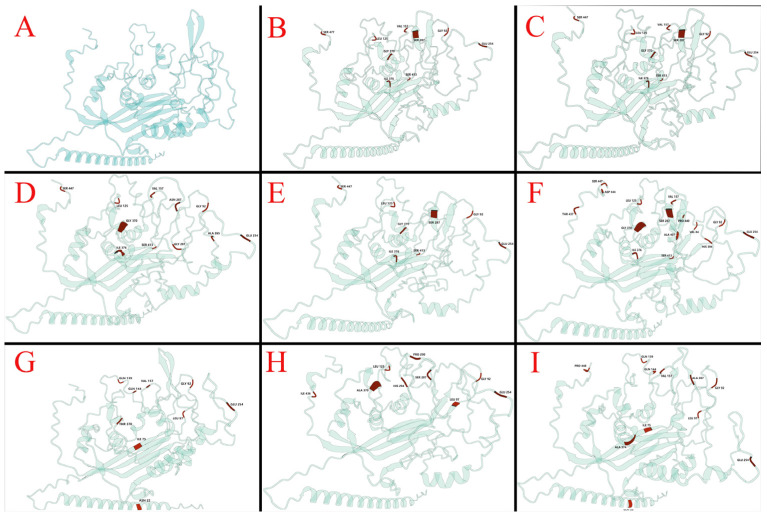
Cartoon representation of CIAV VP1 protein structures. Key amino acid sites are marked (red). (**A**) Reference VP1 protein structure of strain CIAV-YM; (**B**) isolate CIAV-GDHZ1; (**C**) isolate CIAV-GDHZ2; (**D**) isolate CIAV-GDJM; (**E**) isolate CIAV-GDLF; (**F**) reference strain N4; (**G**) reference strain Ahhui1996; (**H**) reference strain JL15120; (**I**) reference strain 98D06073.

**Figure 6:**
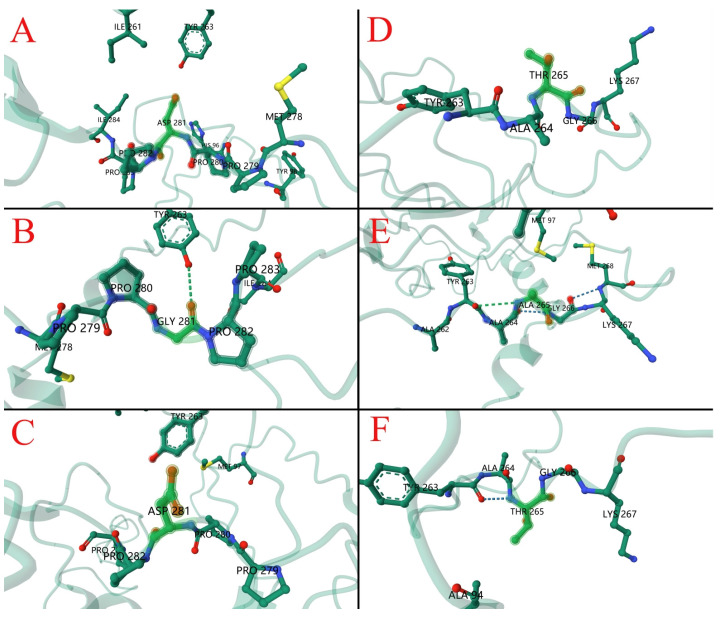
Cartoon representation of local CIAV VP1 protein structures. Key amino acid sites mutation structure are marked (Fluorescent green) (**A**) Local structure at residue 281D of strain CIAV-YM; (**B**) local structure at residue 281G of isolate CIAV-GDJM; (**C**) local structure at residue 281D of isolate CIAV-GDHZ1; (**D**) local structure at residue 265T of strain CIAV-YM; (**E**) local structure at residue 265A of isolate CIAV-GDJM; (**F**) local structure at residue 265T of isolate CIAV-GDHZ1.

Utilizing the Wemol online software (v1.0.3) for Protein Structure Prediction (ESMFold, https://wemol.wecomput.com/ui/#/frontend/workflow/workflow-modules (accessed on 6 June 2024)). According to ESMFold, whose predictions were validated by comparison with known protein structures in the Protein Data Bank (PDB), pLDDT (Per-residue confidence) averaged 34.52 and PTM (Predicted template model) averaged 0.354. Structural predictions of VP1 proteins from vaccine strains, isolates and some reference strains were analyzed and showed that amino acid substitutions resulted in significant structural changes compared to the vaccine strains (Figure 5A–I). In silico analysis of the VP1 protein from the CIAV-GDJM strain revealed a D281G substitution that disrupts the typical beta-sheet secondary structure, introducing an irregular coil conformation in its place (Figure 6). The local protein structure indicated that the D281G substitution formed a hydrogen bond with A263 (Figure 6A–C), while the T265A substitution resulted in a hydrogen bond with Y263 (Figure 6D,E).

## 4. Discussion

China boasts a large-scale poultry industry, incorporating strong breeding and consumption aspects. Notably, Guangdong province has emerged as a leading region in this sector. Guangdong ranks first in China for both poultry inventory and slaughter volume. Furthermore, the province holds the top position nationwide in poultry meat and egg consumption. To maintain a substantial poultry breeding industry, effective disease control measures are of utmost importance. Numerous research studies have investigated the impact of CIAV, a virus that leads to high mortality rates and immunosuppression in avian species. The high prevalence of CIAV as indicated by the high prevalence of CIAV antibodies in chicken has posed a significant challenge to production in China. The epidemiological survey of CIAV in some areas of South China showed that the positive rate of CIAV antibody in chickens in certain areas of Hainan Province in South China was 33% [15]. In comparison, the positive rate of CIAV antibody in chickens in Zhejiang Province was 66.8% [16]. However, the epidemiological characteristics of CIAV in some areas of Guangdong Province are still unclear.

### 4.1. Serological Analysis of CIAV Strains

The high CIAV seroprevalence (79.77%) observed in this study indicates that the virus is widely circulating in Guangdong and remains endemic in both commercial and household chicken populations. Similar findings have been reported in Chinese provinces such as Shandong, Jiangsu, Anhui, and Zhejiang, as well as in India and Bangladesh, where high seroprevalence [13,17,18,19,20,21]. These results underscore the continuing epidemiological challenge posed by CIAV across diverse poultry production systems.

Of particular concern was the detection of seropositivity in all breeder farms, as CIAV is capable of vertical transmission from hens to progeny. Infected breeder populations may therefore act as reservoirs, perpetuating the virus across generations and sustaining endemic circulation at the regional level.

From a prevention and control perspective, these findings highlight the need to strengthen CIAV surveillance and to consider vaccination strategies tailored to local epidemiological conditions. Evidence from genetic and epidemiological studies indicates that mismatches between vaccine and circulating strains can compromise protective efficacy. In line with this, our genetic analyses demonstrated that all Guangdong isolates were genetically distinct from the widely used vaccine strain CIAV-YM, suggesting potential limitations of current immunization practices. These comparisons reinforce the importance of continuous monitoring of CIAV evolution and the development of updated or region-specific vaccines. When combined with rigorous biosecurity and improved breeder stock management, such measures will be essential to mitigate the immunosuppressive and economic impact of CIAV on poultry production.

### 4.2. Molecular Detection Method

The qPCR and conventional PCR assays targeting the VP1 gene developed in this study reliably detected CIAV in thymus samples, as demonstrated by the successful isolation of four viral strains. Compared with previous studies that employed PCR or serological testing alone, our combined approach enhances confidence in identifying true infections [20,22]. A notable discrepancy was observed between the high seroprevalence (79.77%) and the low PCR positivity (4/105). This difference likely reflects the natural course of infection: many seropositive chickens may have recovered from prior CIAV exposure, resulting in reduced thymic viral loads that are difficult to detect by PCR or isolate in vitro. These findings emphasize that serology primarily indicates historical exposure, whereas PCR and virus isolation reveal active infection. Accordingly, integrating multiple diagnostic methods is essential for accurately assessing CIAV prevalence and for informing surveillance strategies.

### 4.3. Isolation of CIAV Strains

Viral isolation using SPF embryonated chicken eggs or MSB1 cells provides a favorable proliferation environment [23,24]. In this study, two methods including SPF embryonated chicken eggs and MSB1 cells were used to isolate the virus. The results indicated that different isolation methods result in differences in CIAV proliferation. Follow-up analysis using PCR showed that the samples were negative for CIAV and that the virus did not proliferate under inoculation of SPF embryonated chicken eggs. The negative result is linked to several factors such as human factors. In addition, these results were attributed to several amino acid substitution. However, studies should explore the effect of virus–host interactions on proliferation outcomes. Microscopic analysis following virus isolation showed morphological changes, including cellular swelling, fragmentation, and other cytopathic changes. Furthermore, indirect immunofluorescence microscopy assay showed green fluorescence in the test group. These findings show that CIAV spread during this study differed significantly in hosts. Further studies should explore the factors that affect virus–host interaction.

### 4.4. Phylogenetic Analysis of CIAV Strains

Phylogenetic analysis of the VP1 gene revealed that all four Guangdong isolates belonged to clade A, consistent with previous reports indicating that this lineage has become predominant in China [13,17,18]. GDHZ1 was positioned within subgroup A1 and showed close genetic relationships with strains reported from eastern China, whereas GDHZ2 clustered within subgroup A2 and was more closely related to isolates from central China. These patterns suggest that CIAV in Guangdong may have been introduced or disseminated from other provinces, consistent with recent studies demonstrating frequent inter-regional genetic connections across China. In contrast, GDJM and GDLF clustered within subgroup A1 together with strains from southern China and eastern Asia, supporting the notion of endemic maintenance in the region. Considering Guangdong’s geographic location in southern China, adjacent to Taiwan and in proximity to Japan, the role of avian migration may also contribute to the introduction and cross-regional dissemination of genetically related CIAV strains [25].

Although the Guangdong isolates exhibited substantial similarity to regional field strains, they were clearly divergent from the widely used commercial vaccine strain CIAV-YM, which belongs to subgroup A4. This genetic separation suggests that circulating field strains have followed distinct evolutionary trajectories compared with the vaccine lineage, raising concerns that current vaccines may provide incomplete protection.

Comparative analyses of the VP1, VP2, and VP3 genes from the isolates, commercial vaccine strain, and reference sequences revealed high overall similarity. The VP1 gene, widely recognized as a marker of CIAV genetic diversity, remains the most informative for sequence analysis. Protein structure predictions indicated that amino acid substitutions within VP1 may induce tertiary structural changes, potentially influencing pathogenicity and antibody binding to antigenic epitopes [26,27].

Previous studies have demonstrated associations between specific VP1 residues and CIAV virulence and replication efficiency. In particular, strains harboring glutamine (Q) at position 394 display greater virulence than those containing histidine (H) at the same position [28]. In this study, all isolates contained glutamine (Q) at position 394 and exhibited the characteristic amino acid profile associated with virulent strains: 75V, 89T, 125L, 139K, 141Q, and 144E (excluding the combined substitutions 75I and 141L) [29,30,31]. These findings indicate that the Guangdong isolates are highly pathogenic and display strong cell tropism, consistent with their robust replication in cell culture.

Of particular note, the CIAV-GDJM strain, isolated from Jiangmen, harbored two novel amino acid substitutions within the VP1 gene at positions 265 (T265A) and 281 (D281G). These substitutions have not been reported in the existing literature. In silico analyses predicted that both substitutions may promote hydrogen bonding with neighboring residues, potentially enhancing protein stability. Such structural modifications may influence viral pathogenicity, replication, and host specificity, warranting further experimental investigation.

## 5. Conclusions

This study combined serological surveillance, molecular detection, and phylogenetic analysis to investigate the epidemiology of CIAV in Guangdong Province between 2018 and 2022. A high seroprevalence (79.77%) was observed across nine cities, indicating widespread circulation of CIAV in both commercial breeding flocks and small-scale rural farms. Phylogenetic analysis of the VP1 gene showed that all four isolates belonged to clade A but separated into different subgroups and were clearly divergent from the commonly used vaccine strain CIAV-YM. Protein analyses further identified amino acid substitutions at virulence-associated sites, suggesting possible structural and functional modifications that could influence pathogenicity and immune recognition.

Collectively, these findings demonstrate that CIAV remains endemic in Guangdong and continues to undergo local evolutionary diversification. They highlight the necessity of sustained epidemiological surveillance and molecular monitoring, as well as the importance of functional studies to clarify the pathogenic and immunological effects of VP1 variation. Such evidence provides a scientific basis for the refinement of control measures and the development of updated or region-specific vaccine strategies.

## 6. Patents

Invention patent: a fluorescent quantitative PCR kit and primers for detection of chicken infectious anemia virus.

## Figures and Tables

**Table 1:** Primers used for quantitative PCR (qPCR) detection of CIAV in this study.

Primer Name	Sequence (5′–3′)	Amplicon Length (bp)
CIAV-124-F	TGCCGGTTCTTTAATCACCC	124 bp
CIAV-124-R	ATCCCTCATTCTTAGTGGCAA	

**Table 2:** Primers used for identification and amplification of full-length CIAV VP1, VP2, and VP3 genes in this study.

Primer Name	Sequence (5′ to 3′)	Amplicon Length (bp)
CIAV-F	GCTCTCCAAGAAGATACTCCA	723 bp
CIAV-R	TATGTTAGGTTCATTGACGCT	
CIAV-F684	AGTAGGTATACGCAAGGCGGT	684 bp
CIAV-R684	AGCCTCACACTATACGTA	
CIAV-F1243	GCGGTATCGTAGACGAGC	1243 bp
CIAV-R1243	CCCTTTTCAGGGCTGCG	

**Table 3:** Number of chicken serum samples collected from different cities in Guangdong Province, 2018–2022.

City	Breed	Number of Serum Samples	Number of CIAV Positive Serum Samples	CIAV Positive Rate of Serum Samples (%)	95% Confidence Interval (CI)	
					Lower CI (%)	Upper CI (%)
Guangzhou	Yangshan chicken	51	47	92.16	81.16	97.04
Foshan	Ma chicken	136	115	84.56	77.44	89.76
Zhanjiang	Three yellow chicken	50	50	100.00	92.89	100.00
Huizhou	Ma chicken	98	67	68.37	58.77	76.60
Qingyuan	Ma chicken	82	70	85.37	76.04	91.51
Jiangmen	Three yellow chicken	162	117	72.22	64.70	78.64
Zhuhai	Yangshan chicken	129	115	89.15	82.40	93.64
Zhongshan	Three yellow chicken	52	20	38.46	26.44	52.31
Shantou	Three yellow chicken	26	26	100	87.06	100

**Table 4:** Details of tissue sample collection from broiler farms.

Farm Name	Breed	Number of Samples	Days-Old of Chickens in the Farm	City	Positive Rate of CAV (Positive Tissue Samples/Total Tissue Samples)	Isolates	Year of Collection
Farm A	Three yellow chicken	12	70	Lufeng	8.33% (1/12)	CIAV-GDLF	2018
Farm B	Ma chicken	10	52	Foshan	0% (0/10)		2018
Farm C	Three yellow chicken	21	65	Huizhou	9.52% (2/21)	CIAV-GDHZ1 CIAV-GDHZ2	2018
Farm D	Ma chicken	15	67	Qingyuan	0% (0/15)		2018
Farm E	Three yellow chicken	17	59	Jiangmen	5.89% (1/17)	CIAV-GDJM	2019
Farm F	Yangshan chicken	13	95	Zhuhai	0% (0/13)		2019
Farm G	Ma chicken	17	83	Shantou	0% (0/17)		2019

**Table 5:** Comparison of key amino acid substitutions in the CIAV VP1 protein among isolates and reference strains.

Strain	Substitution of the Amino Acid Residues in VP1									
	75	89	125	139	141	144	265	281	287	394
CIAV-YM	V	T	I	K	Q	E	T	D	T	Q
CIAV-GDHZ1	-	-	L	-	-	-	-	-	S	-
CIAV-GDHZ2	-	-	L	-	-	-	-	-	S	-
CIAV-JM	-	-	L	-	-	-	A	G	N	-
CIAV-LF	-	-	L	-	-	-	-	-	S	-
N22	I	-	-	Q	-	Q	-	-	-	-
Ahhui1998	I	-	-	Q	-	Q	-	-	-	-
JL15120	-	-	L	-	-	-	-	-	S	-
98D06073	I	-	-	Q	-	Q	-	-	A	-

## Data Availability

The original contributions presented in this study are included in the article/Supplementary Material. Further inquiries can be directed to the corresponding author(s).

## Supplementary Materials

The following supporting information can be downloaded at: https://www.mdpi.com/article/10.3390/vetsci12100972/s1.
