# Supplementary material for: Epidemiological Characteristics and Genetic Diversity of Chicken Infectious Anemia Virus (CIAV) in Guangdong Province, China
Source: Vet Sci. 2025 Oct 10;12(10):972. doi: 10.3390/vetsci12100972 (PMC12567861; doi:10.3390/vetsci12100972)
Supplement: Supplementary file 1 [file vetsci-12-00972-s001.zip › Table S2. Comprehensive Information on CIAV Isolates and Reference Strains.pdf]

Additional file 2. Comprehensive Information on CIAV Isolates and Reference Strains.

No.	Strain name	origin	Accession No.	Time
1	CIAV-GDJM	Guangdong, China	MW817565	2021
2	CIAV-GDLF	Guangdong, China	MW817566	2021
3	CIAV-GDHZ1	Guangdong, China	MW817564	2021
4	CIAV-GDHZ2	Guangdong, China	MW817563	2021
5	JX21614	Guangdong, China	OP038385	2022
6	GD212641	Guangdong, China	OP038343	2022
7	1312PT10	Taiwan, China	KY888915	2017
8	19AD011	South Korea	MW239170	2020
9	GXC060821	Guangxi, China	JX964755	2006
10	N4	Guangdong, China	MK887168	2019
11	C369	Japan	AB046590	2000
12	HN2201	Henan, China	OQ869206	2023
13	CQ21411	Guangdong, China	OP038328	2022
14	HaN211132	Guangdong, China	OP038355	2022
15	FJ211112	Guangdong, China	OP038336	2022
16	JL14026	Heilongjiang, China	KY486147	2017
17	GD-E-12	Guangdong, China	KF224929	2013
18	GD-101	Guangdong, China	KU050680	2015
19	GD-B-12	Guangdong, China	KF224926	2013
20	1401TC03	Taiwan, China	KY888918	2017
21	SH16	Shanghai, China	DQ141671	2005
22	HN9	Henan, China	DQ141672	2005
23	JS15166	Jiangsu, China	KY486153	2015
24	JS2203	Henan, China	OQ869212	2023
25	HN2102	Henan, China	OQ869201	2023
26	JL15120	Jilin, China	KY486149	2015
27	BS-C1	Shandong, China	KX447633	2016
28	HB160430	Shandong, China	KX447635	2016
29	JZ2105	Hubei, China	OQ850272	2023
30	JL190103(VP1)	Jilin, China	MT332124	2020
31	HLJ15170	Heilongjiang, China	KY486144	2016
32	CIAV/IT/CK/1180/19	Italy	MT813076	2020
33	EG-Ismailia-2019	Egypt	MT268631	2020
34	U361402(VP1)	Uruguay	MT118023	2020
35	SMSC-1P60	Malaysia	AF390102	2001
36	17SY0902	Jilin, China	MK089243	2018
37	1709TW	Taiwan, China	MT799756	2020
38	P4(VP1)	Argentina	AJ890284	2005

39	1520TW	Taiwan, China	MK360817	2019
40	AH1998/CHN/2020	Anhui, China	OM355476	2022
41	69(VP1)	Slovenia	DQ016140	2005
42	BD-3	Bangladesh	AF395114	2001
43	HLJ15108	Heilongjiang, China	KY486137	2017
44	Ahhui1998	Anhui, China	OM799843	2022
45	20-SD201911	Shandong, China	OQ116673	2022
46	19AQ001	South Korea	MW091342	2020
47	SD22	Shandong, China	DQ141673	2005
48	SD24	Shandong, China	AY999018	2005
49	CAU269/7	Australia	AF227982	2000
50	3711	Australia	EF683159	2007
51	SMSC-1	Malaysia	AF285882	2001
52	10	Taiwan, China	KJ728821	2012
53	GD-K-12	Guangdong	KF224935	2012
54	98D06073	America	AF311900	2001
55	F10	Shandong, China	KU845735	2015
56	N22	Shandong, China	KU845734	2015
57	SD1403	Shandong, China	KU221054	2014
58	1777TW	Taiwan, China	MN299313	2016
