# Supplementary material for: Epidemiological Characteristics and Genetic Diversity of Chicken Infectious Anemia Virus (CIAV) in Guangdong Province, China
Source: Vet Sci. 2025 Oct 10;12(10):972. doi: 10.3390/vetsci12100972 (PMC12567861; doi:10.3390/vetsci12100972)
Supplement: Supplementary file 1 [file vetsci-12-00972-s001.zip › Table S3. Nucleotide sequence alignment of three proteins among four CIAV isolates and reference strains.pdf]

Additional file3. The nucleotide sequence alignment of three proteins across four CIAV isolates.

Strain	CIAV-GDHZ1			CIAV-GDHZ2			CIAV-GDJM			CIAV-GDLF		
	VP1	VP2	VP3	VP1	VP2	VP3	VP1	VP2	VP3	VP1	VP2	VP3
10	94.9	100	100	95.3	99.7	99.7	94.8	100	100	94.9	99.8	100
69(VP1)	97.6	/	/	97.8	/	/	97.5	/	/	97.8	/	/
1312PT10	99.4	99.8	100	98.5	99.5	99.7	99.1	99.8	100	98.9	99.7	100
1401TC03	98.7	99.8	99.7	99.4	99.5	99.5	98.4	99.8	99.7	98.7	99.7	99.7
1520TW	97.7	99.8	99.7	97.5	99.5	99.5	97.6	99.8	99.7	97.9	99.7	99.7
1709TW	97.6	99.2	98.9	97.3	98.9	98.6	97.5	99.2	98.9	97.8	99.1	98.9
1777TW	94.6	99.4	99.2	95.1	99.1	98.9	94.5	99.4	99.2	94.7	99.2	99.2
17SY0902	98.4	99.5	99.5	98.3	99.2	99.2	98.2	99.5	99.5	98.6	99.4	99.5
19AD011	98.4	99.5	99.7	98.3	99.2	99.5	98.1	99.5	99.7	98.6	99.4	99.7
19AQ001	97.6	99.7	99.7	97.5	99.4	99.5	97.5	99.7	99.7	97.9	99.5	99.7
20-SD201911	97.6	99.7	100	97.4	99.4	99.7	97.5	99.7	100	97.8	99.8	100
3711	95.3	99.1	98.6	94.9	98.8	98.4	94.9	99.1	98.6	95.0	99.1	98.6
98D06073	94.4	99.8	100	94.9	99.5	99.7	94.3	99.8	100	94.4	99.7	100
AH1998/CHN/2020	97.7	99.8	99.7	97.6	99.5	99.5	97.4	99.8	99.7	97.9	99.7	99.7
Ahhui1998	97.6	99.8	100	97.5	99.5	99.7	97.5	99.8	100	97.9	99.7	100

BD-3	97.3	99.8	99.7	97.2	99.5	99.5	97.3	99.8	99.7	97.6	99.7	99.7
BS-C1	97.3	99.8	99.7	97.5	99.5	99.5	97.0	99.8	99.7	97.7	99.7	99.7

Continued Supplementary materials

Strain	CIAV-GDHZ1			CIAV-GDHZ2			CIAV-GDJM			CIAV-GDLF		
	VP1	VP2	VP3	VP1	VP2	VP3	VP1	VP2	VP3	VP1	VP2	VP3
C369	99.0	100	100	98.7	99.7	99.7	98.7	100	100	99.0	99.8	100
CAU269/7	95.2	99.1	98.6	94.7	98.8	98.4	94.9	99.1	98.6	94.9	99.1	98.6
CIAV/IT/CK/1180/19	97.6	99.5	99.5	97.6	99.2	99.2	97.6	99.5	99.5	97.7	99.4	99.5
CQ21411	98.7	99.4	99.5	98.4	99.1	99.2	98.3	99.4	99.5	99.1	99.5	99.5
EG-Ismailia-2019	97.6	99.4	99.2	97.5	99.1	98.9	97.5	99.4	99.2	97.6	99.2	99.2
F10	94.4	98.6	99.2	94.7	98.6	98.9	94.3	98.6	99.2	94.5	98.5	99.2
FJ211112	98.9	99.7	100	98.4	99.4	99.7	98.4	99.7	100	99.7	99.8	100
GD-101	98.7	99.5	99.2	98.5	99.2	98.9	98.4	99.2	99.2	99.0	99.4	99.2
GD-B-12	98.7	99.5	99.2	98.5	99.2	98.9	98.4	99.2	99.2	99.0	99.4	99.2
GD-E-12	98.6	99.8	100	98.4	99.5	99.7	98.3	99.8	100	99.0	99.7	100
GD-K-12	95.3	99.2	98.9	95.4	98.9	98.6	95.0	99.2	98.9	95.1	99.1	98.9
GD212641	99.3	99.5	99.5	98.4	99.2	99.2	99.0	99.5	99.5	98.7	99.4	99.5
GXC060821	99.0	99.5	99.7	98.7	99.2	99.5	98.7	99.5	99.7	99.0	99.4	99.7
HaN211132	99.0	99.8	100	98.5	99.5	99.7	98.5	99.8	100	99.8	100	100
HB160430	97.4	99.7	99.5	97.6	99.4	99.2	97.1	99.7	99.5	97.8	99.5	99.5

HLJ15108	97.6	99.8	100	97.5	99.5	99.7	97.5	99.8	100	97.6	99.7	100
HLJ15170	97.2	99.8	100	97.5	99.5	99.7	97.0	99.8	100	97.6	99.7	100

Continued Supplementary materials

Strain	CIAV-GDHZ1			CIAV-GDHZ2			CIAV-GDJM			CIAV-GDLF		
	VP1	VP2	VP3	VP1	VP2	VP3	VP1	VP2	VP3	VP1	VP2	VP3
HN2102	97.4	100	100	97.7	99.7	99.7	97.2	100	100	97.8	99.8	100
HN2201	98.7	99.8	100	98.2	99.5	99.7	98.2	99.8	100	99.0	99.7	100
HN9	98.4	99.8	99.7	98.2	99.5	99.5	98.1	99.8	99.7	98.6	99.7	99.7
JL14026	98.4	99.9	99.7	98.3	99.5	99.5	98.1	99.8	99.7	98.8	99.7	99.7
JL15120	97.3	100	100	97.4	99.7	99.7	97.0	100	100	97.6	99.8	100
JL190103(VP1)	97.2	/	/	97.5	/	/	97.0	/	/	97.6	/	/
JS15166	98.4	100	100	98.2	99.7	99.7	98.1	100	100	98.6	99.8	100
JS2203	98.1	99.8	100	97.9	99.5	99.7	97.7	99.8	100	98.4	100	100
JX21614	98.9	100	100	98.0	99.7	99.7	98.6	100	100	98.4	99.8	100
JZ2105	97.3	100	100	97.5	99.7	99.7	97.1	100	100	97.6	99.8	100
N22	94.2	99.1	98.9	94.6	98.8	98.6	94.1	99.1	98.9	94.3	98.9	98.9
N4	98.4	99.4	99.5	98.4	99.1	99.2	98.1	99.4	99.5	98.7	99.2	99.5
P4(VP1)	97.6	/	/	97.3	/	/	97.5	/	/	97.8	/	/
SD1403	94.4	99.2	99.2	95.0	98.9	98.9	94.4	99.2	99.2	94.6	99.1	99.2
SD22	97.6	99.7	99.7	97.6	99.4	99.5	97.3	99.7	99.7	97.6	99.5	99.7

SD24	97.3	99.8	99.7	97.3	99.5	99.5	97.0	99.8	99.7	97.3	99.7	99.7
SH16	98.6	100	100	99.0	99.7	99.7	98.3	100	100	98.8	99.8	100

Continued Supplementary materials

Strain	CIAV-GDHZ1			CIAV-GDHZ2			CIAV-GDJM			CIAV-GDLF		
	VP1	VP2	VP3	VP1	VP2	VP3	VP1	VP2	VP3	VP1	VP2	VP3
SMSC-1	94.7	99.8	100	95.0	99.5	99.7	94.6	99.8	100	94.7	99.7	100
SMSC-1P60	97.6	99.8	99.7	97.4	99.5	99.5	97.5	99.8	99.7	97.9	99.7	99.7
U361402(VP1)	97.6	/	/	97.5	/	/	97.5	/	/	97.9	/	/
