# Supplementary material for: Epidemiological Characteristics and Genetic Diversity of Chicken Infectious Anemia Virus (CIAV) in Guangdong Province, China
Source: Vet Sci. 2025 Oct 10;12(10):972. doi: 10.3390/vetsci12100972 (PMC12567861; doi:10.3390/vetsci12100972)

Additional file 4. Amino acid sequence alignment of the VP1 gene among CIAV isolates and reference strains.

		20		40		60		
CIAV-YM	MARRARRPRG	RFYAFRRGRW	HHLKRLRRRY	KFRHRRRQRY	RRRAFRKAFH	NPRPGTYSVR	LPNPQSTMTI	70
CIAV-GDHZ1	....	....	....	....	....	....	....	70
CIAV-GDHZ2	....	....	....	....	....	....	....	70
CIAV-GDJM	....	....	....	....	....	....	....	70
CIAV-GDLF	....	....	....	....	....	....	....	70
GXC060821	....	....	....	....	....	....	....	70
GD-E-12	....	....	....	....	....	....	....	70
GD-B-12	....	....	....	....	....	....	....	70
GD-K-12	....	....	.Q........	....	....	....	....	70
GD-101	....	....	....	....	....	....	....	70
SH16	....	....	....	....	....	....	....	70
1312PT10	....	....	....	....	....	....	....	70
JL14026	....	....	....	....	....	....	....	70
SD22	....	....	....	....	....	....	....	70
JX21614	....	....	....	....	....	....	....	70
FJ211112	....	....	....	....	....	....	....	70
3711	....	....	....	....	....	....	....	70
19AD011	....	....	....	....	....	....	....	70
JS15166	....	....	....	....	....	....	....	70
HN9	....	....	....	....	....	....	....	70
1401TC03	....	....	....	....	....	....	....	70
SD24	....	....	....	....	....	....	....	70
U361402	....	....	.N........	....	....	....	....	70
17SY0902	....	....	.N........	....	....	....	....	70
GD212641	....	....	....	....	....	....	....	70
C369	....	....	....	....	....	....	....	70
HN2201	....	....	....	....	....	....	....	70
HaN211132	....	....	....	....	....	....	....	70
EG-Ismailia-2019	....	....	.Q........	....	....	....	....	70
CIAV/IT/CK/1180/19	....	....	.Q........	....	....	....	....	70
P4	....	....	....	....	....	....	....	70
1709TW	....	....	....	....	....	....	....	70
1520TW	....	....	....	....	....	....	....	70
SMSC-1P60	....	....	....	....	....	....	....	70
JS2203	....	....	....	....	....	....	....	70
JZ2105	....	....	....	....	....	....	....	70
JL190103	....	....	....	....	....	....	....	70
BS-C1	....	....	....	....	....	....	....	70
JL15120	....	....	....	....	....	....	....	70
HN2102	....	....	....	....	....	....	....	70
HLJ15170	....	....	.Y........	....	....	....	....	70
HB160430	....	....	....	....	....	....	....	70
CAU269/7	....	....	....	....	....	....	....	70
HLJ15108	....	....	.N........	....	....	....	....	70
19AQ001	....	....	.N........	....	....	....	....	70
69	....	....	....	....	....	....	....	70
20-SD201911	....	....	.N........	....	....	....	....	70
98D06073	....	....	.Q........	....	....	....	....	70
BD-3	....	....	....	....	....	....	....	70
Ahhui1998	....	....	.N........	....	....	....	....	70
N22	....	....	.Q........	....	....	....	....	70
1777TW	....	....	.Q........	....	....	....	....	70
SMSC-1	....	....	....	....	....	....	....	70
10	....	....	....	....	....	....	....	70
AH1998/CHN/2020	....	....	....	....	....	....	..D.......	70
SD1403	....	....	.Q........	....	....	....	....	70
N4	....	....	....	....	....	....	....	70
CQ21411	....	....	....	....	....	....	....	70
F10	..I.T.....	....	LR........	....	....	....	....	70
Consensus	MARRARRPRG	RFYAFRRGRW	HHLKRLRRRY	KFRHRRRQRY	RRRAFRKAFH	NPRPGTYSVR	LPNPQSTMTI	

Conservation 100% 0%

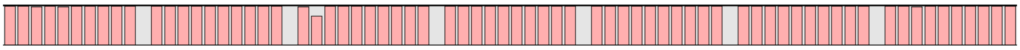

```
                                        80                            100                           120                           140
CIAV-YM                RFQGVIFLTE GLILPKNSTA GDYADHMYGA RVAKISVNLK EFLLASMNLT YVSKIGGPIA GELIADGSKS 140
CIAV-GDHZ1             .......... .......... .G........ .......... .......... ....L..... .......... 140
CIAV-GDHZ2             .......... .......... .G........ .......... .......... ....L..... .......... 140
CIAV-GDJM              .......... .......... .G........ .......... .......... ....L..... .......... 140
CIAV-GDLF              .......... .......... .G........ .......... .......... ....L..... .......... 140
GXC060821              .......... .......... .G........ .......... .......... ....L..... .......... 140
GD-E-12                .......... .......... .G........ .......... .......... ....L..... .......... 140
GD-B-12                .......... .......... .G........ .......... .......... ....L..... .......... 140
GD-K-12                ....I..... .......... .G....L... .......... .......... .......... ........Q. 140
GD-101                 .......... .......... .G........ .......... .......... ....L..... .......... 140
SH16                   .......... .......... .G........ .......... .......... ....L..... .......... 140
1312PT10               .......... .......... .G........ .......... .......... ....L..... .......... 140
JL14026                .......... .......... .G........ .......... .......... ....L..... .......... 140
SD22                   .......... .......... .G........ .......... .......... ....L..... .......... 140
JX21614                .......... .......... .G........ .......... .......... ....L..... .......... 140
FJ211112               .......... .......... .G........ .......... .......... ....L..... .......... 140
3711                   .......... .......... .G........ .......... .......... .......... .......... 140
19AD011                .......... .......... .G........ .......... .......... ....L..... .......... 140
JS15166                .......... .......... .G........ .......... .......... ....L..... .......... 140
HN9                    .......... .......... .G........ .......... .......... ....L..... .......... 140
1401TC03               .......... .......... .G........ .......... .......... .A..L..... .......... 140
SD24                   .......... .......... .G........ .......... .......... ....L..... .......... 140
U361402                .......... .......... .G........ .......... .......... .......... .......... 140
17SY0902               .......... .......... .G........ .......... .......... .......... .......... 140
GD212641               .......... .......... .G..N..... .......... .......... ....L..... .......... 140
C369                   .......... .......... .G........ .......... .......... ....L..... .........A 140
HN2201                 .......... .......... .G........ .......... .......D.. ....L..... .......... 140
HaN211132              .......... .......... .G........ .......... .......... ....L..... .......P.. 140
EG-Ismailia-2019       .......... .......... .G........ .......... .......... .......... .......... 140
CIAV/IT/CK/1180/19     .......... .......... .G........ .......... .......... .......... .......... 140
P4                     .......... .......... .......... .......... .......... .......... .......... 140
1709TW                 .......... .......... .......... .......... .......... .......... .......... 140
1520TW                 .......... .......... .G........ .......... .......... .......... .......... 140
SMSC-1P60              .......... ..L....... .G........ .......... .......... .......... .......... 140
JS2203                 .......... .......... .G........ .......... .......... ....L..... .......... 140
JZ2105                 .......... .......... .G....L... .......... .......... ....L..... .......... 140
JL190103               .......... .......... .G....L... .......... .......... ....L..... .......... 140
BS-C1                  .......... .......... .G....L... .......... .......... ....L..... .......... 140
JL15120                .......... .......... .G....L... .......... .......... ....L..... .......... 140
HN2102                 .......... .......... .G........ .......... .......... ....P..... .......... 140
HLJ15170               .......... .......... .G....L... .......... .......... ....L..... .......... 140
HB160430               .......... .......... .G....L... .......... .......... ....L..... .......... 140
CAU269/7               .......... .......... .G........ .......... .......... .......... .......... 140
HLJ15108               ....I..... .......... .G....L... .......... .......... .......... ........Q. 140
19AQ001                ....I..... .......... .G....L... .......... .......... .......... ........Q. 140
69                     ....I..... .......... .G....L... .......... .......... .......... ........Q. 140
20-SD201911            ....I..... .......... .G....L... .......... .......... .......... ........Q. 140
98D06073               ....I..... .......... .G....L... .......... .......... .......... ........Q. 140
BD-3                   ....I..... .......... .G....L... .......... .......... .......... ........Q. 140
Ahhui1998              ....I..... .......... .G....L... .......... .......... .......... ........Q. 140
N22                    ....I..... .......... .G....L... .......... .......... .......... ........Q. 140
1777TW                 ....I..... .......... .G....L... .......... .......... .......... ........Q. 140
SMSC-1                 ....I..... .......... .E....L... .......... .......... .......... ........Q. 140
10                     ....I..... .......... .G....L... .......... .......... .......... ........Q. 140
AH1998/CHN/2020        .......... .......... .G....L... .......... .......... .......... ........Q. 140
SD1403                 ....I..... .......... .G....L... .......... .......... .......... ........Q. 140
N4                     .......... ...V...... .G........ .......... .......... ....L..... .......... 140
CQ21411                .......... .......... .G........ .......... .......... ....L..... .......... 140
F10                    ....I..... .......... .G....L... .......... .......... .......... ........Q. 140
Consensus              RFQGVIFLTE GLILPKNSTA GGYADHMYGA RVAKISVNLK EFLLASMNLT YVSKLGGPIA GELIADGSKS
```

Conservation

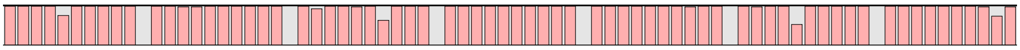

```
                                      160         170         180         190         200
                                        |                       |                       |
CIAV-YM                QAAENWPNCW LPLDNNMPSA TPSAWWRWAL MMMQPTDSCR FFNHPKQMTL QDMGRMFGGW HLFRHIETRF 210
CIAV-GDHZ1             .......... ......V... .......... .......... .......... .......... .......... 210
CIAV-GDHZ2             .......... ......V... .......... .......... .......... .......... .......... 210
CIAV-GDJM              .......... ......V... .......... .......... .......... .......... .......... 210
CIAV-GDLF              .......... .......... .......... .......... .......... .......... .......... 210
GXC060821              .......... ......V... .......... .......... .......... .......... .......... 210
GD-E-12                .......... .......... .......... .......... .......... .......... .......... 210
GD-B-12                .......... .......... .......... .......... .......... .......... .......... 210
GD-K-12                ...Q...... ......V... .......... .......... .......... .......... .......... 210
GD-101                 .......... .......... .......... .......... .......... .......... .......... 210
SH16                   .......... ......V... .......... .......... .......... .......... .......... 210
1312PT10               .......... ......V... .......... .......... .......... .......... .......... 210
JL14026                .......... .......... .......... .......... .......... .......... .......... 210
SD22                   .......... ......V... .......... .......... .......... .......... .......... 210
JX21614                .......... ......V... .......... .......... .......... .......... .......... 210
FJ211112               .......... .......... .......... .......... .......... .......... .......... 210
3711                   .......... ......V... .......... .......... .......... .......... .......... 210
19AD011                .......... .......... .......... .......... .......... .......... .......... 210
JS15166                .......... ......V... .......... .......... .......... .......R.. .......... 210
HN9                    .......... ......V... .......... .......... .......... .......R.. .......... 210
1401TC03               .......... ......V... .......... .......... .......... .......... .......... 210
SD24                   .......... ......V... .......... .......... ..Y....... .......... .......... 210
U361402                .......... ......V... .......... .......... .......... .......... .......... 210
17SY0902               .......... ......V... .......... .......... .......... .......... .......... 210
GD212641               .......... ......V... .......... .......... .......... .......... .......... 210
C369                   .......... ......V... .......... .......... .......... .......... .......... 210
HN2201                 .......... .......... .......... .......... .......... .......... .......... 210
HaN211132              .......... .......... .......... .......... .......... .......... .......... 210
EG-Ismailia-2019       ........M. ......V... .......... .......... .......... .......... .......... 210
CIAV/IT/CK/1180/19     ........M. ......V... .......... .......... .......... .......... .......... 210
P4                     .......... .......... .......... .......... .......... .......... .......... 210
1709TW                 .......... .......... .......... .......... .......... .......... .......... 210
1520TW                 .......... .......... .......... .......... .......... .......... .......... 210
SMSC-1P60              E......... .......... .......... .......... .......... .......... .......... 210
JS2203                 .......... .......... .......... .......... .......... .......... .......... 210
JZ2105                 .......... .......... .......... .......... .......... .......... .......... 210
JL190103               .......... .......... .......... .......... .......... .......... .......... 210
BS-C1                  .......... .......... .......... .......... .......... .......... .......... 210
JL15120                .......... .......... .......... .......... .......... .......... .......... 210
HN2102                 .......... .......... .......... .......... .......... .......... .......... 210
HLJ15170               .......... .......... .......... .......... .......... .......... .......... 210
HB160430               .......... .......... .......... .......... .......... .......... .......... 210
CAU269/7               .......... ......V... .......... .......... .......... .......... .......... 210
HLJ15108               ...Q...... ......V... .......... .......... .......... .......... .......... 210
19AQ001                ...Q...... ......V... .......... .......... .......... .......... .......... 210
69                     ...Q...... ......V... .......... .......... .......... .......... .......... 210
20-SD201911            ...Q...... ......V... .......... .......... .......... .......... .......... 210
98D06073               ...Q...... ......V... .......... .......... .......... .......... .......... 210
BD-3                   ...Q...... ......V... .......... .......... .......... .......... .......... 210
Ahhui1998              ...Q...... ......V... .......... .......... .......... .......... .......... 210
N22                    ...Q...... ......V... .......... .......... .......... .......... .......... 210
1777TW                 ...Q...... ......V... .......... .......... .......... .......... .......... 210
SMSC-1                 ...Q...... ......V... .......... .......... .......... .......... .......... 210
10                     ...Q...... ......V... .......... .......... .......... .......... .......... 210
AH1998/CHN/2020        .......... ......V... .......... .......... .......... .......... .......... 210
SD1403                 ...Q...... ......V... .......... .......... .......... .......... .......... 210
N4                     .......... ......V... .......... .......... .......... .......... .......... 210
CQ21411                .......... .......... .......... .......... .......... .......... .......... 210
F10                    ...Q...... ......V... .......... .......... .......... .......... .......... 210
Consensus              QAAENWPNCW LPLDNNVPSA TPSAWWRWAL MMMQPTDSCR FFNHPKQMTL QDMGRMFGGW HLFRHIETRF
```

Conservation 100% 0%

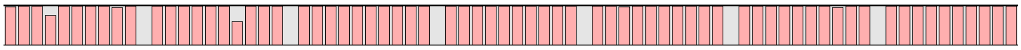

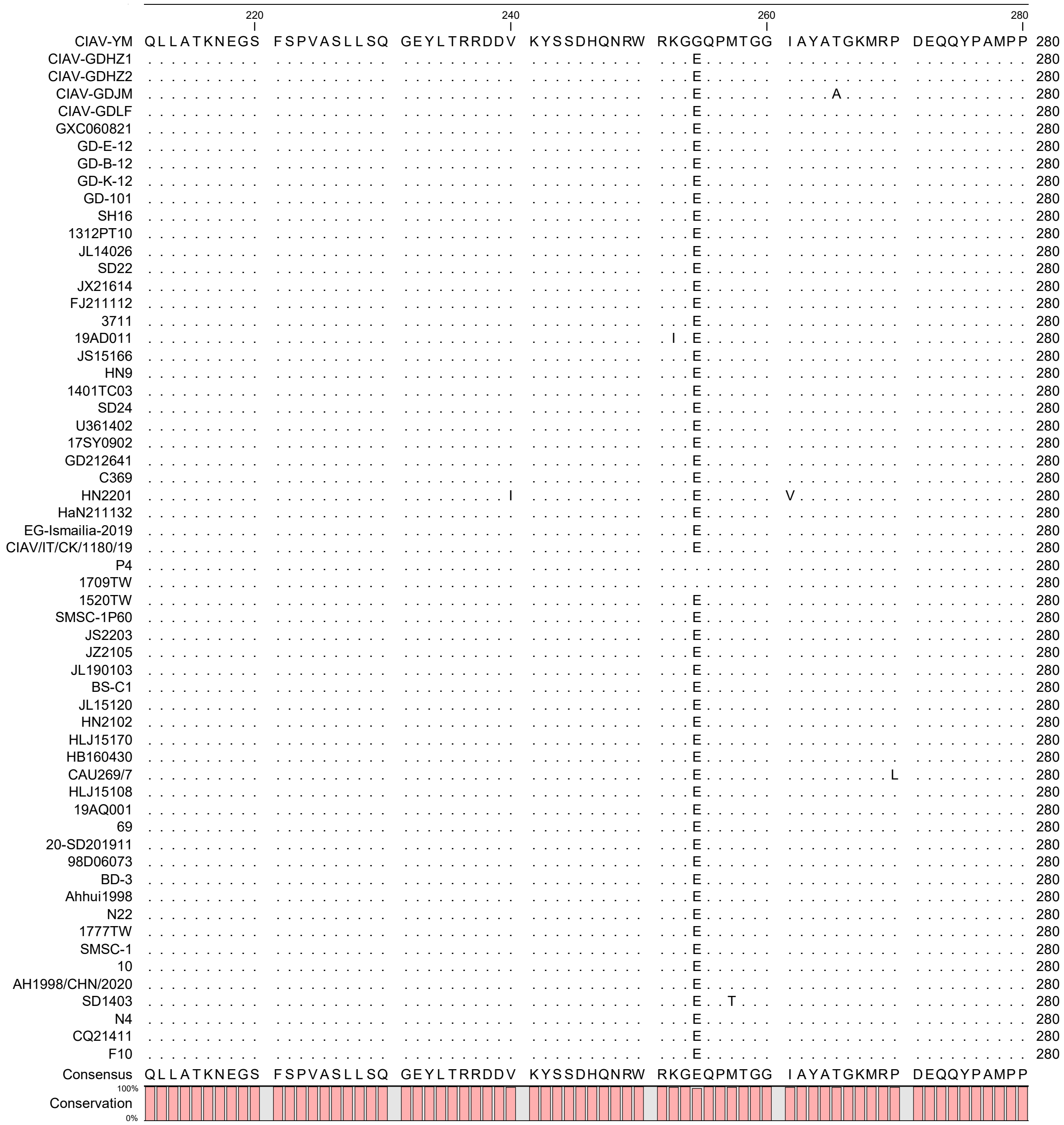
220 240 260 280
CIAV-YM QLLATKNEGS FSPVASLLSQ GEYLTRRDDV KYSSDHQNRW RKGGQPMTGG IAYATGKMRP DEQQYPAMPP 280
CIAV-GDHZ1 ....E...... 280
CIAV-GDHZ2 ....E...... 280
CIAV-GDJM ....E...... ....A..... 280
CIAV-GDLF ....E...... 280
GXC060821 ....E...... 280
GD-E-12 ....E...... 280
GD-B-12 ....E...... 280
GD-K-12 ....E...... 280
GD-101 ....E...... 280
SH16 ....E...... 280
1312PT10 ....E...... 280
JL14026 ....E...... 280
SD22 ....E...... 280
JX21614 ....E...... 280
FJ211112 ....E...... 280
3711 ....E...... 280
19AD011 .... .I.E...... 280
JS15166 ....E...... 280
HN9 ....E...... 280
1401TC03 ....E...... 280
SD24 ....E...... 280
U361402 ....E...... 280
17SY0902 ....E...... 280
GD212641 ....E...... 280
C369 ....E...... 280
HN2201 ....I ....E...... V......... 280
HaN211132 ....E...... 280
EG-Ismailia-2019 ....E...... 280
CIAV/IT/CK/1180/19 ....E...... 280
P4 280
1709TW 280
1520TW ....E...... 280
SMSC-1P60 ....E...... 280
JS2203 ....E...... 280
JZ2105 ....E...... 280
JL190103 ....E...... 280
BS-C1 ....E...... 280
JL15120 ....E...... 280
HN2102 ....E...... 280
HLJ15170 ....E...... 280
HB160430 ....E...... 280
CAU269/7 ....E...... ....L 280
HLJ15108 ....E...... 280
19AQ001 ....E...... 280
69 ....E...... 280
20-SD201911 ....E...... 280
98D06073 ....E...... 280
BD-3 ....E...... 280
Ahhui1998 ....E...... 280
N22 ....E...... 280
1777TW ....E...... 280
SMSC-1 ....E...... 280
10 ....E...... 280
AH1998/CHN/2020 ....E...... 280
SD1403 ....E..T... 280
N4 ....E...... 280
CQ21411 ....E...... 280
F10 ....E...... 280
Consensus QLLATKNEGS FSPVASLLSQ GEYLTRRDDV KYSSDHQNRW RKGEQPMTGG IAYATGKMRP DEQQYPAMPP
100%
Conservation
0%

```
                                        300                   320                   340
                                          |                     |                     |
CIAV-YM              DPPIITTTTA QGTQVRCMNS TQAWWSWDTY MSFATLTALG AQWSFPPGQR SVSRRSFNHH KARGAGDPKG 350
CIAV-GDHZ1           ......S... .......... .......... .......... .......... .......... .......... 350
CIAV-GDHZ2           ......S... .......... .......... .......... .......... .......... .......... 350
CIAV-GDJM            G.....N... .......... .......... .......... .......... .......... .......... 350
CIAV-GDLF            ......S... .......... .......... .......... .......... .......... .......... 350
GXC060821            ......S... .......... .......... .......... .......... .......... .......... 350
GD-E-12              ......S... .......... .......... .......... .......... .......... .......... 350
GD-B-12              ......S... .......... .......... .......... .......... .......... .......... 350
GD-K-12              ......A... .......... .......... .......... .......... .......... .......... 350
GD-101               ......S... .......... .......... .......... .......... .......... .......... 350
SH16                 ......S... .......... .......... .......... .......... .......... .......... 350
1312PT10             ......S... .......... .......... .......... .......... .......... .......... 350
JL14026              ......S... .......... .......... .......... .......... .......... .......... 350
SD22                 ......S... .......... .......... .......... .......... .......... .......... 350
JX21614              ......S... .......... .......... .......... .......... .......... .......... 350
FJ211112             ......S... .......... .......... .......... .......... .......... .......... 350
3711                 ......S... .......... .......... .......... .......... .......... .......... 350
19AD011              ......S... .......... .......... .......... .......... .......... .......... 350
JS15166              ......S... .......... .......... .......... .......... .......... .......... 350
HN9                  ......S... .......... .......... .......... .......... .......... .......... 350
1401TC03             ......S... .......... .......... .......... .......... .......... .......... 350
SD24                 ......S... .......... .......... .......... .......... .......... .......... 350
U361402              ......S... .......... .......... .......... .......... .......... .......... 350
17SY0902             ......S... .......... .......... .......... .......... .......... .......... 350
GD212641             ......S... .......... .......... .......... .......... .......... .......... 350
C369                 ......S... .......... .......... .......... .......... .......... .......... 350
HN2201               ......S... .......... .......... .......... .......... .......... .......... 350
HaN211132            ......S... .......... .......... .......... .......... .........R .......... 350
EG-Ismailia-2019     ......S... .......... .......... .......... .......... .......... .......... 350
CIAV/IT/CK/1180/19   ......S... .......... .......... .......... .......... .......... .......... 350
P4                   .......... .......... .......... .......... .......... .......... .......... 350
1709TW               .......... .......... .......... .......... .......... .......... .......... 350
1520TW               .......... .......... .......... .......... .......... .......... .......... 350
SMSC-1P60            ......S... ......Y... .......... .......... .......... .......... .......... 350
JS2203               .........P ...H...... .......... .......... .......... .......... .......... 350
JZ2105               .........P ...H...... .......... .......... .......... .......... .......... 350
JL190103             .........P ...H...... .......... .......... .......... .......... .......... 350
BS-C1                ......S..P ...H...... .......... .......... .......... .......... .......... 350
JL15120              ......S..P ...H...... .......... .......... .......... .......... .......... 350
HN2102               .........P ...H...... .......... .......... .......... .......... .......... 350
HLJ15170             .........P ...H...... .......... .......... .......... .......... .......... 350
HB160430             ......S..P .......... .......... .......... .......... .......... .......... 350
CAU269/7             .......... .......... .......... .......... .......... .......... .......... 350
HLJ15108             ......A... .......... .......... .......... .......... .......... .......... 350
19AQ001              ......A... .......... .......... .......... .......... .......... .......... 350
69                   ......A... .......... .......... .......... .......... .......... .......... 350
20-SD201911          ......A... .......... .......... .......... .......... .......... .......... 350
98D06073             ......A... .......... .......... .......... .......... .......... .......... 350
BD-3                 ......A... .......... .......... .......... .......... .......... .......... 350
Ahhui1998            .......... .......... .......... .......... .......... .......... .......... 350
N22                  .......QPP ..P....... .......... .......... .......... .......... .......... 350
1777TW               .........P .......... .......... .......... .......... .......... .......... 350
SMSC-1               .........P ...P...... .......... .......... .......... .......... .......... 350
10                   .........P .......... .......... .......... .......... .......... .......... 350
AH1998/CHN/2020      ......A... .......... .......... .......... .......... .......... .......... 350
SD1403               .........P .......... .......... .......... .......... .......... .......... 350
N4                   ......S... .......... .......... .......... .......... .......... .......... 350
CQ21411              ......S... .......... .......... .......... .......... .......... .......... 350
F10                  .......AL. .......... .......... .......... .......... .......... .......... 350
Consensus            DPPIITSTTA QGTQVRCMNS TQAWWSWDTY MSFATLTALG AQWSFPPGQR SVSRRSFNHH KARGAGDPKG
```

Conservation

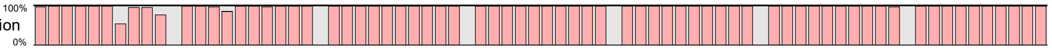

```
                      360        380        400        420
CIAV-YM               QRWHTLVPLG TETITDSYMS APASELDTNF FTLYVAQGTN KSQQYKFGTA TYALKEPVMK SDAWAVVRVQ 420
CIAV-GDHZ1            .......... .........G .....I.... .......... .......... .......... ..S....... 420
CIAV-GDHZ2            .......... .........G .....I.... .......... .......... .......... ..S....... 420
CIAV-GDJM             .......... .........G .....I.... .......... .......... .......... ..S....... 420
CIAV-GDLF             .......... .........G .....I.... .......... .......... .......... ..S....... 420
GXC060821             .......... .........G .....I.... .......... .......... .......... ..S....... 420
GD-E-12               .......... .........G .....I.... .......... .......... .......... ..S....... 420
GD-B-12               .......... .........G .....I.... .......... .......... .......... ..S....... 420
GD-K-12               .......... .......... .......... .......... .......... .......... .......... 420
GD-101                .......... .........G .....I.... .......... .......... .......... ..S....... 420
SH16                  .......... .........G .......... .......... .......... .......... ..S....... 420
1312PT10              .......... .........G .....I.... .......... .......... .......... ..S....... 420
JL14026               .......... .........G .....I.... .......... .......... .......... ..S....... 420
SD22                  .......... .......... .......... .......... .......... .......... .......... 420
JX21614               .......... .........G .....I.... .......... .......... .......... ..S....... 420
FJ211112              .......... .........G .....I.... Y......... .......... .......... ..S....... 420
3711                  .......... .........G .......... .......... .......... .......... ..S....... 420
19AD011               .......... .........G .....I.... .......... .......... .......... ..S....... 420
JS15166               .......... .........G .......... .......... .......... .......... ..S....... 420
HN9                   .......... .........G .......... .......... .......... .......... ..S....... 420
1401TC03              .......... .........G .....I.... .......... .......... .......... ..S....... 420
SD24                  .......... .......... .......... .......... .......... .......... .......... 420
U361402               .......... .........G .......... .......... .......... .......... ..S....... 420
17SY0902              .......... .........G .......... .......... .......... .......... ..S....... 420
GD212641              .......... .........G .....I.... .......... .......... .......... ..S....... 420
C369                  .......... .........G .....I.... .......... ...H...... .......... ..S....... 420
HN2201                .......... .........G .....I.... .......... .......... .......... ..S....... 420
HaN211132             .......... .........G .....I.... .......... .......... .......... ..S....... 420
EG-Ismailia-2019      .......... .........G .......... .......... .......... .......... ..S....... 420
CIAV/IT/CK/1180/19    .......... .........G .......... .......... .......... .......... ..S....... 420
P4                    .......... .......... .......... .......... .......... .......... .......... 420
1709TW                .......... .......... .......... .......... .......... .......... .......... 420
1520TW                .......... .......... .......... .......... .......... .......... .......... 420
SMSC-1P60             .......... .........G .......... .......... .......... .......... .......... 420
JS2203                .......... .........R .......... .......... .......... .......... .......... 420
JZ2105                .......... .........A .......... .......... .......... .......... .......... 420
JL190103              .......... .........A .......... .......... .......... .......... .......... 420
BS-C1                 .......... .........A .......... .......... .......... .......... .......... 420
JL15120               .......... .........A .......... .......... .......... .......... .......... 420
HN2102                .......... .........A .......... .......... .......... .......... .......... 420
HLJ15170              .......... .........A .......... .......... .......... .......... .......... 420
HB160430              .......... .........A .......... .......... .......... .......... .......... 420
CAU269/7              .......... .........R .......... .......... .......... .......... ..S....... 420
HLJ15108              .......... .......... .......... .......... .......... .......... .......... 420
19AQ001               .......... .......... .......... .......... .......... .......... .......... 420
69                    .......... .........T .......... .......... .......... .......... .......... 420
20-SD201911           .......... .......... .......... .......... .......... .......... ......L... 420
98D06073              .......... .......... ...A...... .......... .......... .......... .......... 420
BD-3                  .......... .........T .......... .......... .......... .......... .......... 420
Ahhui1998             .......... .........T .......... .......... .......... .......... .......... 420
N22                   .......... .......... .......... .......... .......... .......... .......... 420
1777TW                .......... .......... .......... .......... .......... .......... .......... 420
SMSC-1                .......... .......... .......... .......... .......... .......... .......... 420
10                    .......... .......... .......... .......... .......... .......... .......... 420
AH1998/CHN/2020       .......... .......... .......... .......... .......... .......... .......... 420
SD1403                .......... .........T ....K..... .......... .......... .......... .......... 420
N4                    .......... .........G .....I.... .......... ...H.....P ......A... ..S....... 420
CQ21411               .......... .........G .....I.A.. .......... .......... .......... ..S......P 420
F10                   .......... .......... .......... .......... .......... .......... .......... 420
Consensus             QRWHTLVPLG TETITDSYMG APASELDTNF FTLYVAQGTN KSQQYKFGTA TYALKEPVMK SDAWAVVRVQ
```

Conservation

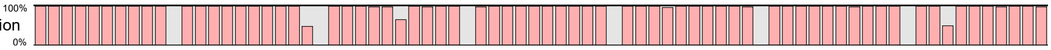

```
                                      440
                                        |
CIAV-YM               SVWQLGNRQR PYPWDVNWAN STMYWGTQP* 450
CIAV-GDHZ1            .......... .......... ......S... 450
CIAV-GDHZ2            .......... .......... ......S... 450
CIAV-GDJM             .......... .......... ......S... 450
CIAV-GDLF             .......... .......... ......S... 450
GXC060821             .......... .......... ......S... 450
GD-E-12               .......... .......... ......S... 450
GD-B-12               .......... .......... ......S... 450
GD-K-12               .......... .......... ......S... 450
GD-101                .......... .......... ......S... 450
SH16                  .......... .......... .......... 450
1312PT10              .......... .......... .......... 450
JL14026               .......... .......... .......... 450
SD22                  .......... .......... ......S... 450
JX21614               .......... .......... .N.C..SET. 450
FJ211112              .......... .......... ......S... 450
3711                  .......... .......... ......S... 450
19AD011               .......... .......... ......S... 450
JS15166               .......... .......... .......... 450
HN9                   .......... .......... .......... 450
1401TC03              .......... .......... .......... 450
SD24                  .......... .......... ......S... 450
U361402               .......... .......... ......G... 450
17SY0902              .......... .......... ......S... 450
GD212641              .......... .......... ......W.T. 450
C369                  .......... .......... ...D..S... 450
HN2201                .......... .......... ......S... 450
HaN211132             .......... .......... ......SH.. 450
EG-Ismailia-2019      .......... .......... ......G... 450
CIAV/IT/CK/1180/19    .......... .......... ......S... 450
P4                    .......... .......... .......... 450
1709TW                .......... .......... .......... 450
1520TW                .......... .......... .......... 450
SMSC-1P60             .......... .......... .......... 450
JS2203                .......... .......... ......S... 450
JZ2105                .......... .....I.... ......S... 450
JL190103              .......... .....I.... ......S... 450
BS-C1                 .......... .....I.... ......S... 450
JL15120               .......... .....I.... .......... 450
HN2102                .......... .....I.... ......S... 450
HLJ15170              .......... .....I.... ......S... 450
HB160430              .......... .....I.... ......S... 450
CAU269/7              .......... .......... ......S... 450
HLJ15108              .......... .......... ......S... 450
19AQ001               .......... .......... ......S... 450
69                    .......... .......... ......S... 450
20-SD201911           .......... .......... ......S... 450
98D06073              .......... .......... ......SP.. 450
BD-3                  .......... .......... .......... 450
Ahhui1998             .......... .......... .......... 450
N22                   .......... .......... ......S... 450
1777TW                .......... .......... .......... 450
SMSC-1                .......... .......... ......S... 450
10                    .......... .......... ......S... 450
AH1998/CHN/2020       .......... .......... ......SH.. 450
SD1403                .......... .......... ......S... 450
N4                    .......... ......T... ...D..S... 450
CQ21411               ...HP.T..T ....V..... ......S... 450
F10                   .......... .......... ......PH.. 450
Consensus             SVWQLGNRQR PYPWDVNWAN STMYWGSQPX
```

Conservation

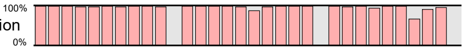

Supplement: Supplementary file 1 [file vetsci-12-00972-s001.zip › Table S4. Amino acid sequence alignment of the VP1 gene among CIAV isolates and reference strains.pdf]
