# Supplementary material for: Epidemiological Characteristics and Genetic Diversity of Chicken Infectious Anemia Virus (CIAV) in Guangdong Province, China
Source: Vet Sci. 2025 Oct 10;12(10):972. doi: 10.3390/vetsci12100972 (PMC12567861; doi:10.3390/vetsci12100972)
Supplement: Supplementary file 1 [file vetsci-12-00972-s001.zip › Table S6. Confidence assessment of VP1 protein structure prediction results (ESMFold).pdf]

Additional file 6. Confidence assessment of VP1 protein structure prediction results (ESMFold)

Strain	Per-residue confidence(pLDDT)	Predicted template model(PTM)
GDHZ1	34.097	0.348
GDHZ2	34.097	0.348
GDJM	33.845	0.341
GDLF	33.985	0.348
YM	35.951	0.375
98D06073	34.146	0.348
Ahhui1998	36.279	0.385
JL15120	34.068	0.349
N4	34.214	0.342

Notes: pLDDT values range from 0-100, with larger values indicating higher structural reliability, and pTM values range from 0-1, with larger values indicating higher structural reliability.
