# Supplementary material for: Epidemiological Characteristics and Genetic Diversity of Chicken Infectious Anemia Virus (CIAV) in Guangdong Province, China
Source: Vet Sci. 2025 Oct 10;12(10):972. doi: 10.3390/vetsci12100972 (PMC12567861; doi:10.3390/vetsci12100972)
Supplement: Supplementary file 1 [file vetsci-12-00972-s001.zip › Date S5. Invention patent. a fluorescent quantitative PCR kit and primers for detection of chicken infectious anemia virus.pdf]

Additional file 5. Invention patent :a fluorescent quantitative PCR kit and primers for detection of chicken infectious anemia virus

Patent name: A fluorescent quantitative PCR kit and primers for detection of chicken infectious anemia virus

Patent application number: 202111219887.X

Issuing date: 2021.10.21

International Patent Classification(Int.Cl.): C12N 15/11(2006.01); C12Q 1/70(2006.01); C12Q 1/6851(2018.01)

Invention content:

(1) The objective of this invention is to provide a rapid detection system for avian infectious anemia virus using quantitative fluorescent PCR, in order to address one or more technical issues present in existing technologies, and to offer at least one beneficial alternative or create conditions.

(2) The present invention provides a quantitative fluorescent PCR kit for detecting avian infectious anemia virus, including the primer pair CIAV-124-F and CIAV-124-R.(Table S1)

Table S1. qPCR primers used in this partent.

Primer name	Sequence (5’ - 3’)	Amplicon length (bp)
CIAV-124-F	TGCCGGTTCTTTAATCACCC	124bp
CIAV-124-R	ATCCCTCATTCTTAGTGGCAA	

(3) The total volume of the detection system described is 20μL, specifically consisting of: 10μL of SYBR Green Premix Ex Taq, 0.5μL of CIAV-124-F, 0.5 μL of CIAV-124-R, 1μL of detection template, and 8μL of ddH2O. The reaction program is as follows: maintain at 95°C for 2 minutes; maintain at 95°C for 30 seconds,

62°C for 20 seconds, and 72°C for 30 seconds, repeat for 40 cycles.

(4) The standard curve equation for the aforementioned quantitative fluorescent PCR kit is: Y = -3.2514X + 37.091, with a correlation coefficient R^2 of 0.9995; the amplification efficiency E is 103.03%.(Figure S9)

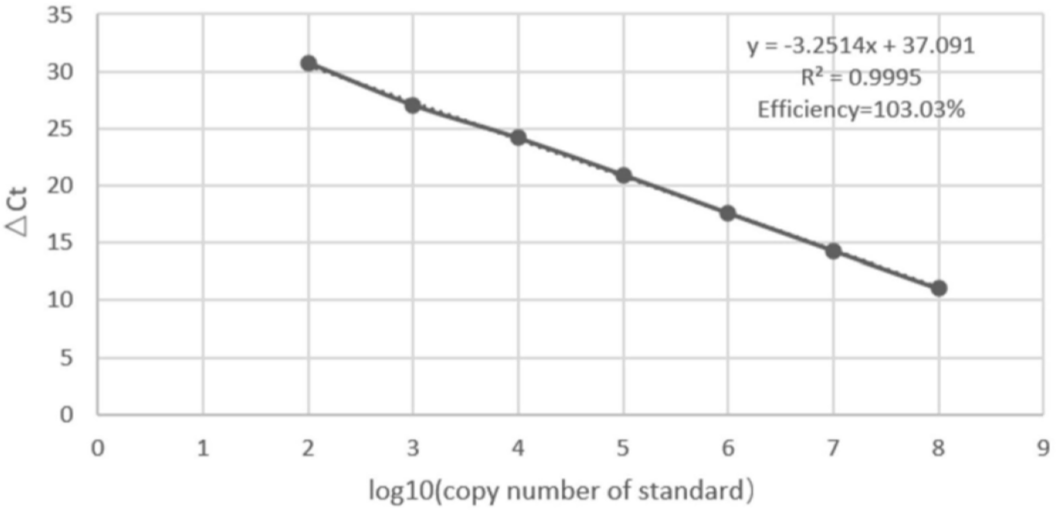

Figure S9. Standard curve of CIAVreal-time fluorescent quantitative PCR kit

(5) The present invention provides primer pairs that can specifically amplify highly conserved gene fragments in the VP1 gene of CIAV, effectively enhancing the anti-interference ability of molecular biology detection for CIAV. Experimental results confirm that the detection limit of the described quantitative fluorescent PCR kit is 2.0 x 10^1 copies/μL, which is 1000 times more sensitive than the minimum detection limit of 2.0 x 10^-1 copies/μL for conventional PCR(Figure S10 Figure S11). Moreover, the kit exhibits good specificity, with no significant amplification curves observed for common avian diseases such as AIV-5, AIV-7, ILT, NDV, IBV, and Fadv-4(Figure S12, Figure S13).

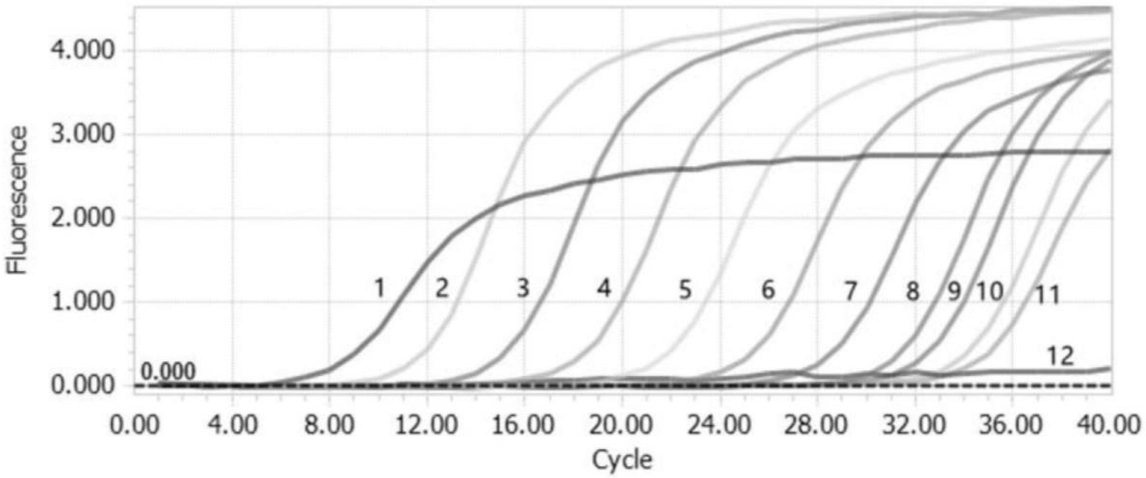

Figure S10. Sensitivity test amplification results of qPCR. Notes: M.500 DNA Marker; 1~12: 2.0×10^{9}～2.0×10^{-1}copies/μL standard plasmid; 12: Negative control.

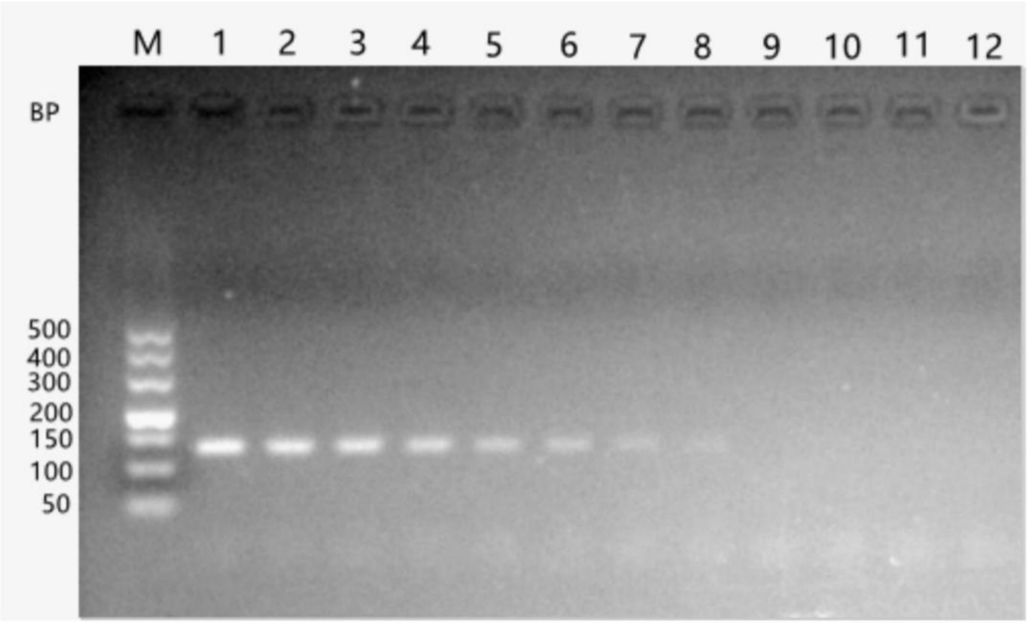

Figure S11. Sensitivity test amplification results of conventional PCR. Notes: M.500 DNA Marker; 1~12: 2.0×10^{9}～2.0×10^{-1}copies/μL standard plasmid; 12: Negative control.

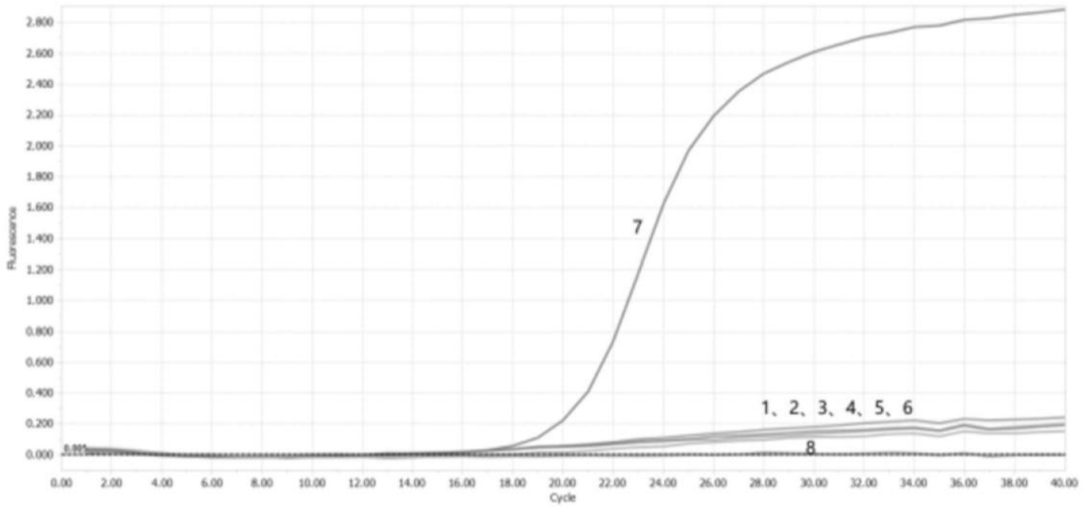

Figure S12. Specificity test results amplification curves.

Patent front page: 1: AIV-5; 2: AIV-7; 3: ILT; 4: NDV; 5: IBV; 6: Fadv-4; 7: CIAV; 8: Negative control.

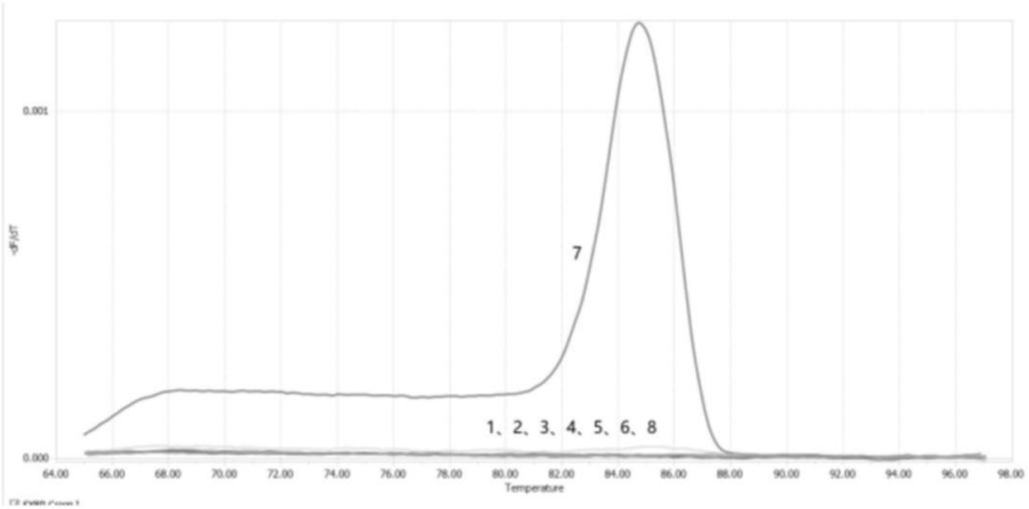

Figure S13. Specificity test results mleting curves.

Patent front page: 1: AIV-5; 2: AIV-7; 3: ILT; 4: NDV; 5: IBV; 6: Fadv-4; 7: CIAV; 8: Negative control.
