# Supplementary material for: Epidemiological Characteristics and Genetic Diversity of Chicken Infectious Anemia Virus (CIAV) in Guangdong Province, China
Source: Vet Sci. 2025 Oct 10;12(10):972. doi: 10.3390/vetsci12100972 (PMC12567861; doi:10.3390/vetsci12100972)
Supplement: Supplementary file 1 [file vetsci-12-00972-s001.zip › Figure S1. PCR results of chicken thymus.pdf]

Additional file 1. PCR results of chicken thymus.

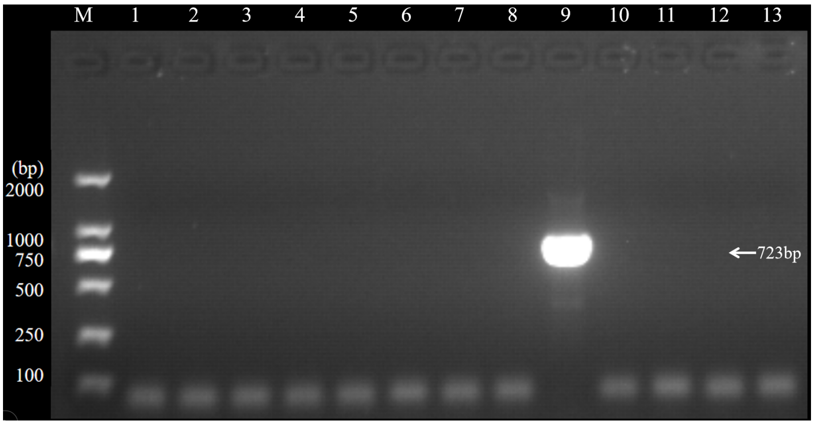

Figure S1. PCR results of thymus samples in Lufeng

M. Trans2K DNA Marker; 1: Negative control; 2~13. Thymus samples LF1~LF12.

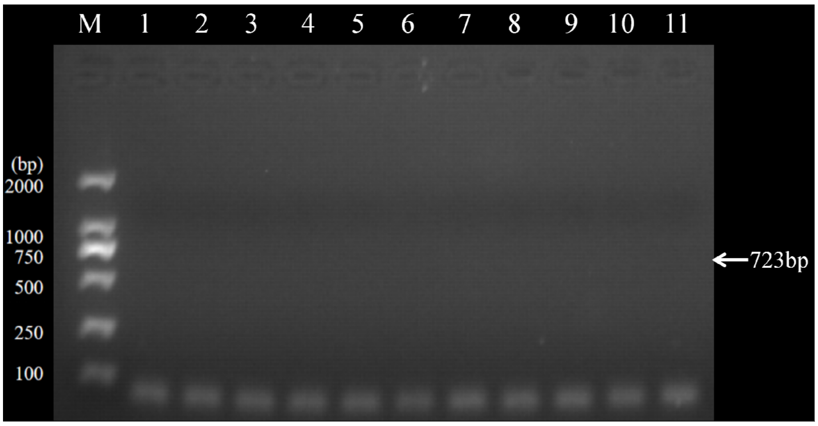

Figure S2. PCR results of thymus samples in Foshan

M. Trans2K DNA Marker; 1: Negative control; 2~11. Thymus samples FS1~LF10.

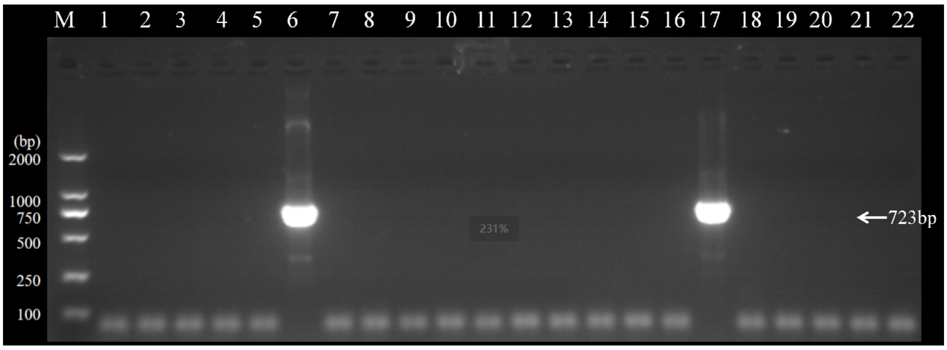

Figure S3. PCR results of thymus samples in Huizhou

M. Trans2K DNA Marker; 1: Negative control; 2~22. Thymus samples HZ1~HZ21.

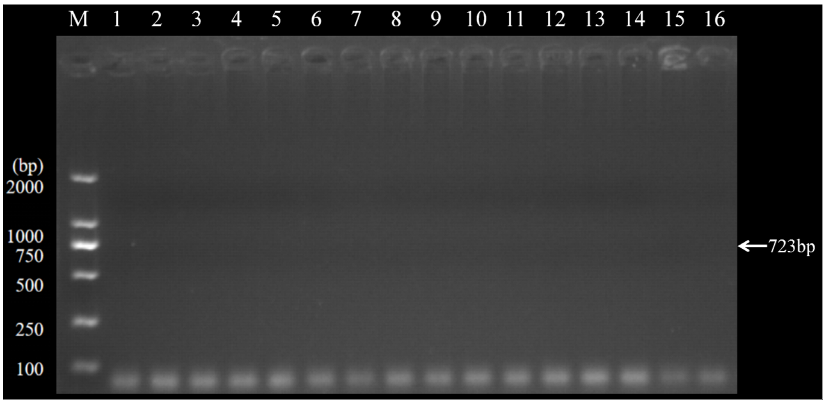

Figure S4. PCR results of thymus samples in Qingyuan

M.Trans2K DNA Marker; 1: Negative control; 2~16. Thymus samples QY1~QY15.

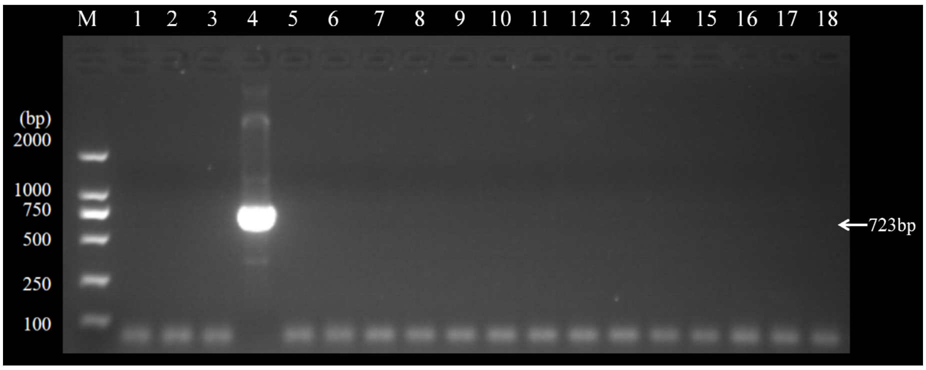

Figure S5. PCR results of thymus samples in Jiangmen

M.Trans2K DNA Marker; 1: Negative control; 2~18. Thymus samples JM1~JM17.

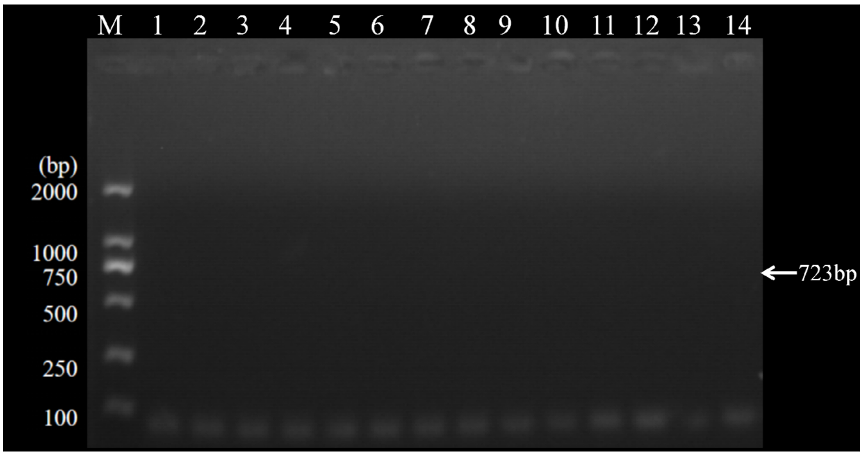

Figure S6. PCR results of thymus samples in Zhuhai

M.DNA Marker; 1: Negative control; 2~14. Thymus samples ZH1~ZH13.

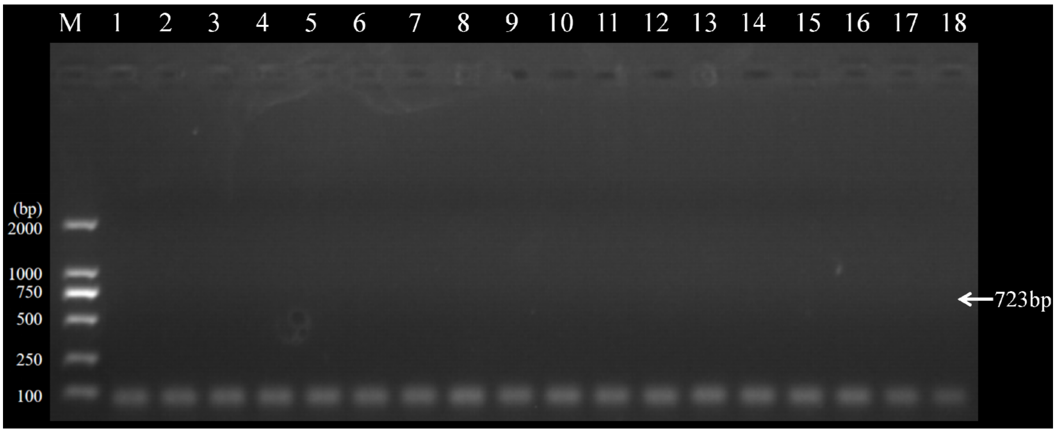

Figure S7. PCR results of thymus samples in Shantou

M. DNA Marker; 1: Negative control; 2~17. Thymus samples ST1~ST13.
