# Supplementary figures and images for: Epidemiological Characteristics and Genetic Diversity of Chicken Infectious Anemia Virus (CIAV) in Guangdong Province, China
Source: Vet Sci. 2025 Oct 10;12(10):972. doi: 10.3390/vetsci12100972 (PMC12567861; doi:10.3390/vetsci12100972)

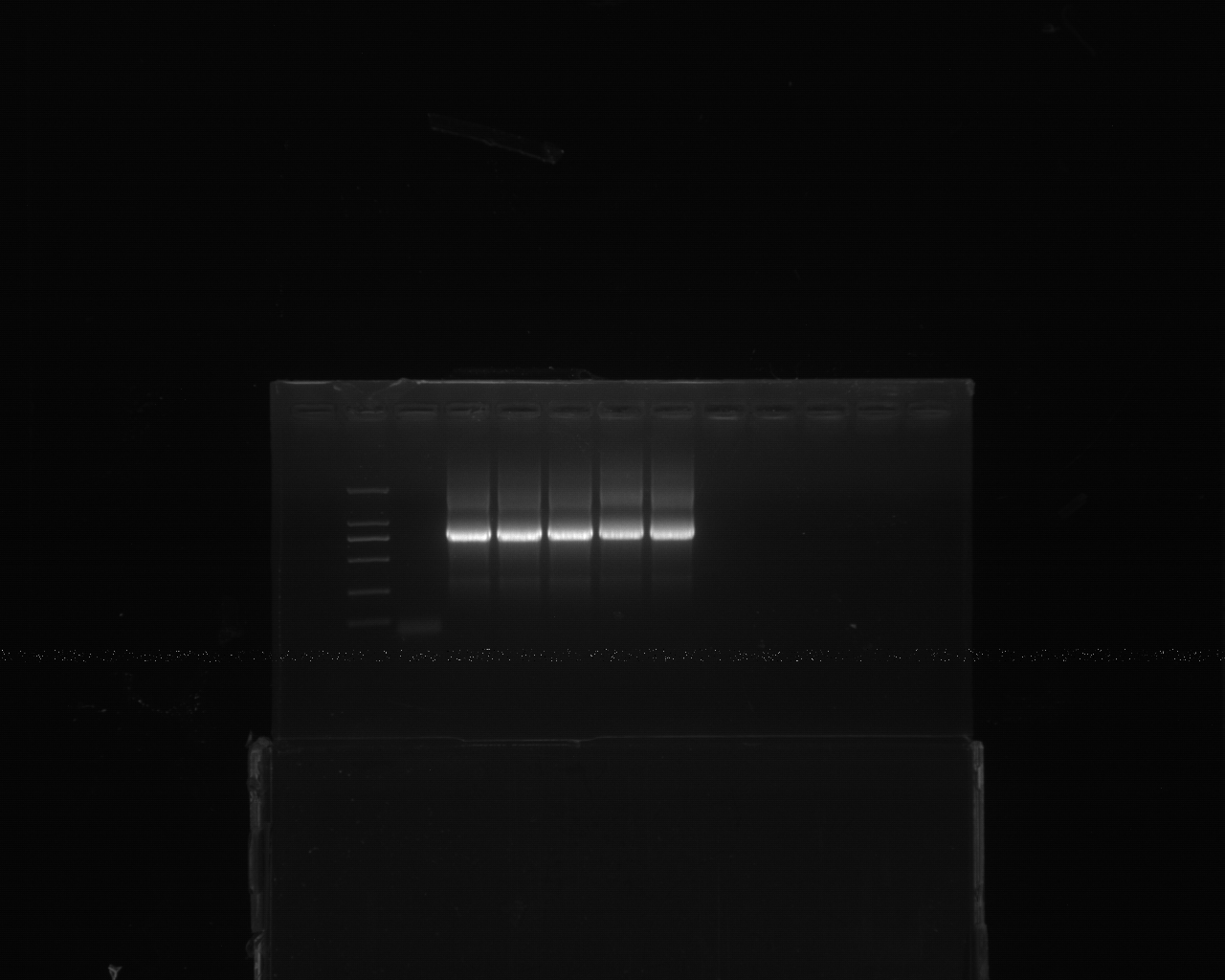

Supplement: Supplementary file 1 [file vetsci-12-00972-s001.zip › Figure S8. Original PCR amplification image of the CIAV VP1 gene from selected thymus tissue samples.Tif]
